# Supplementary material for: ROCK inhibition suppresses host blastomere-mediated elimination and enhances trophoblast stem cell contribution in mouse chimeras
Source: Stem Cell Reports. 2026 Oct 1;21(10):103111. doi: 10.1016/j.stemcr.2026.103111 (PMC13681403; doi:10.1016/j.stemcr.2026.103111)
Supplement: Supplementary file 1 [file mmc1.pdf]

Stem Cell Reports, Volume *21*

Supplemental Information

ROCK inhibition suppresses host blastomere-mediated elimination and enhances trophoblast stem cell contribution in mouse chimeras

Baoxing Dong, Kuisheng Liu, Chenxiang Xi, Sheng Wang, Yifan Sheng, Jiani Xiang, Jiayu Chen, Yingdong Liu, Dandan Bai, Shanyao Liu, Xia Tu, Xiaochen Kou, Yanhong Zhao, Jiqing Yin, Hong Wang, Binya Liu, Changle Ma, Shaorong Gao, and Wenqiang Liu

Supplemental Information

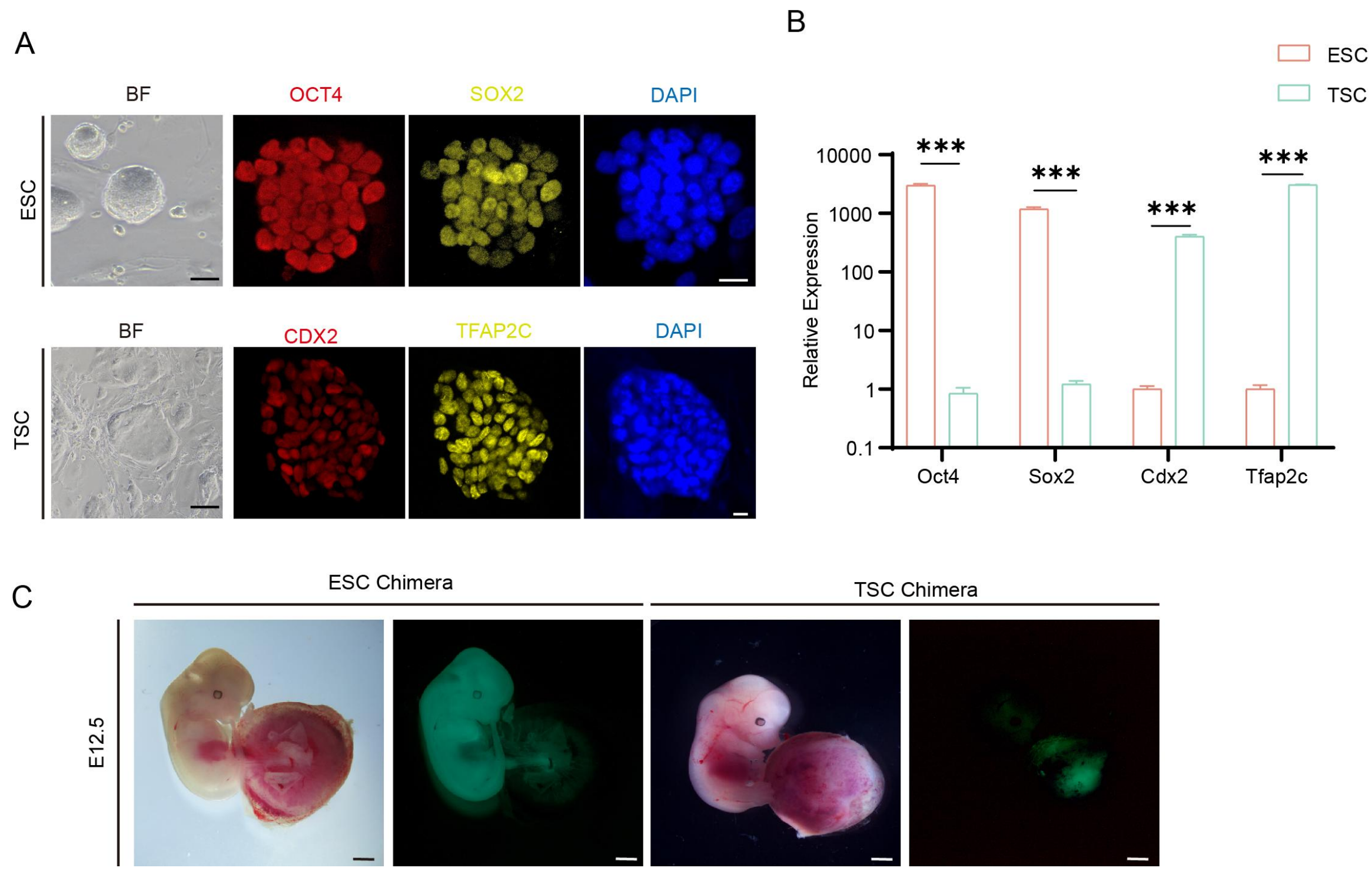

Figure S1. Characterization and In Vivo Contribution Potential of Derived Stem Cell Lines (Related to Figure 1)

(A) Immunofluorescence characterization of donor cell lines. ESC clones were stained for pluripotency markers OCT4 and SOX2; TSC clones were stained for trophoblast markers CDX2 and TFAP2C. Scale bar = 25 μm.

(B) Quantitative PCR (qPCR) analysis of pluripotency and trophoblast lineage-specific markers in derived ESC and TSC lines, with expression levels normalized to the internal reference gene Gapdh. Data are presented as mean ± SD. ***$P < 0.001$, determined by unpaired two-tailed Student's t-test. Data were analyzed using the $2^{\wedge -\Delta\Delta CT}$ method.

(C) Morphology of E12.5 chimeras. Representative bright-field and fluorescence images of embryos following ESC or TSC injection. Scale bar = 2 mm.

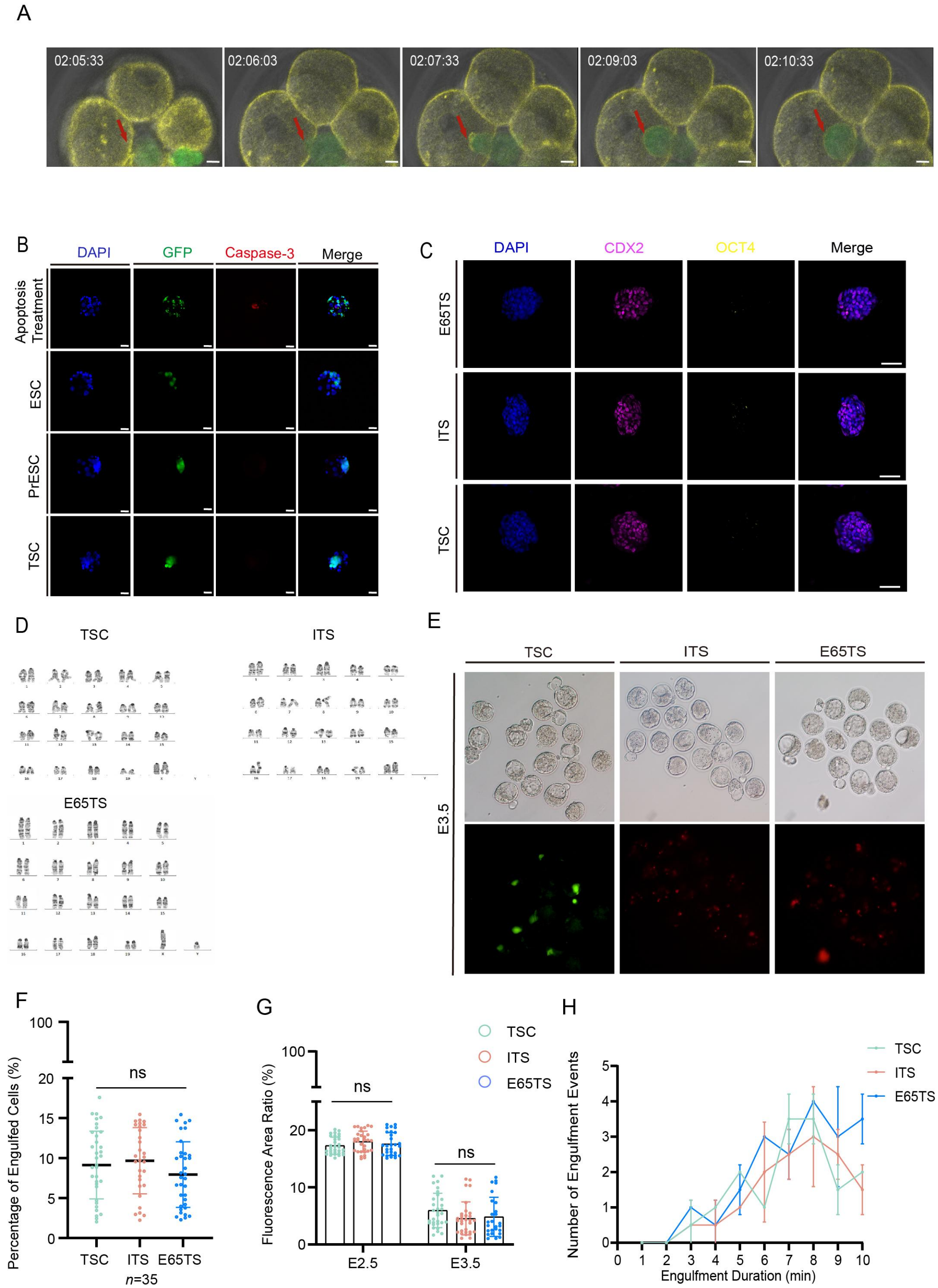
A
02:05:33
02:06:03
02:07:33
02:09:03
02:10:33
B
DAPI
GFP
Caspase-3
Merge
Apoptosis Treatment
ESC
PrESC
TSC
C
DAPI
CDX2
OCT4
Merge
E65TS
ITS
TSC
D
TSC
ITS
E65TS
E
TSC
ITS
E65TS
E3.5
F
Percentage of Engulfed Cells (%)
100
20
15
10
5
0
ns
TSC
ITS
E65TS
n=35
G
Fluorescence Area Ratio (%)
100
20
10
0
ns
ns
E2.5
E3.5
TSC
ITS
E65TS
H
Number of Engulfment Events
5
4
3
2
1
0
0 1 2 3 4 5 6 7 8 9 10
Engulfment Duration (min)
TSC
ITS
E65TS

Fig. S2 Characterization of TSC Internalization Across Multiple Cell Lines. (Related to Figure 2)

(A) Dynamics of phagocytic engulfment. Time-lapse imaging showing the active internalization of donor cells (green) by host blastomeres (yellow, actin). Red arrows indicate donor cells undergoing cell engulfment.

(B) Apoptosis assessment of chimeric embryos. Immunofluorescence staining for Caspase-3 in ESC, PrESC, and TSC chimeras at 4 hours post-injection, showing negligible apoptosis in donor cells. Scale bar = 5 μm.

(C) Immunofluorescence of diverse TSC lines. Characterization of ITS (pre-implantation origin) and E65TS (post-implantation origin) lines using CDX2 and OCT4 markers to confirm TSC identity.

(D) Karyotype analysis. Representative karyotypes of TSC, ITS, and E65TS lines, demonstrating chromosomal stability.

(E) Representative brightfield and fluorescence images of chimeric embryos at the late blastocyst (E3.5) stage.

(F) Representative images of chimeric blastocysts. Comparison of engulfment events among TSC, ITS, and E65TS donor groups at E3.5. Data are presented as mean ± SD. not significant, determined by one-way ANOVA followed by Tukey's post-hoc test. n=42 for TSC, 39 for ITS, and 45 for E65TS (N=3 independent experiments).

(G) Quantitative analysis of TSC line engulfment. Statistics showing the percentage of engulfed cells and the fluorescence area ratio for TSC, ITS, and E65TS at the morula (E2.5) and blastocyst (E3.5) stages. Data are presented as mean ± SD. ns, not significant, determined by one-way ANOVA followed by Tukey's post-hoc test. n=35 for TSC, 37 for ITS, and 32 for E65TS (N=3 independent experiments).

(H) Time distribution statistics of phagocytic events. The number distribution of phagocytic events occurring in the three cell lines (TSC, ITS, and E65TS) at different time points. Temporal distribution and duration of engulfment events across different

TSC lines. Data were recorded from N=20 embryos and processed using GraphPad Prism.

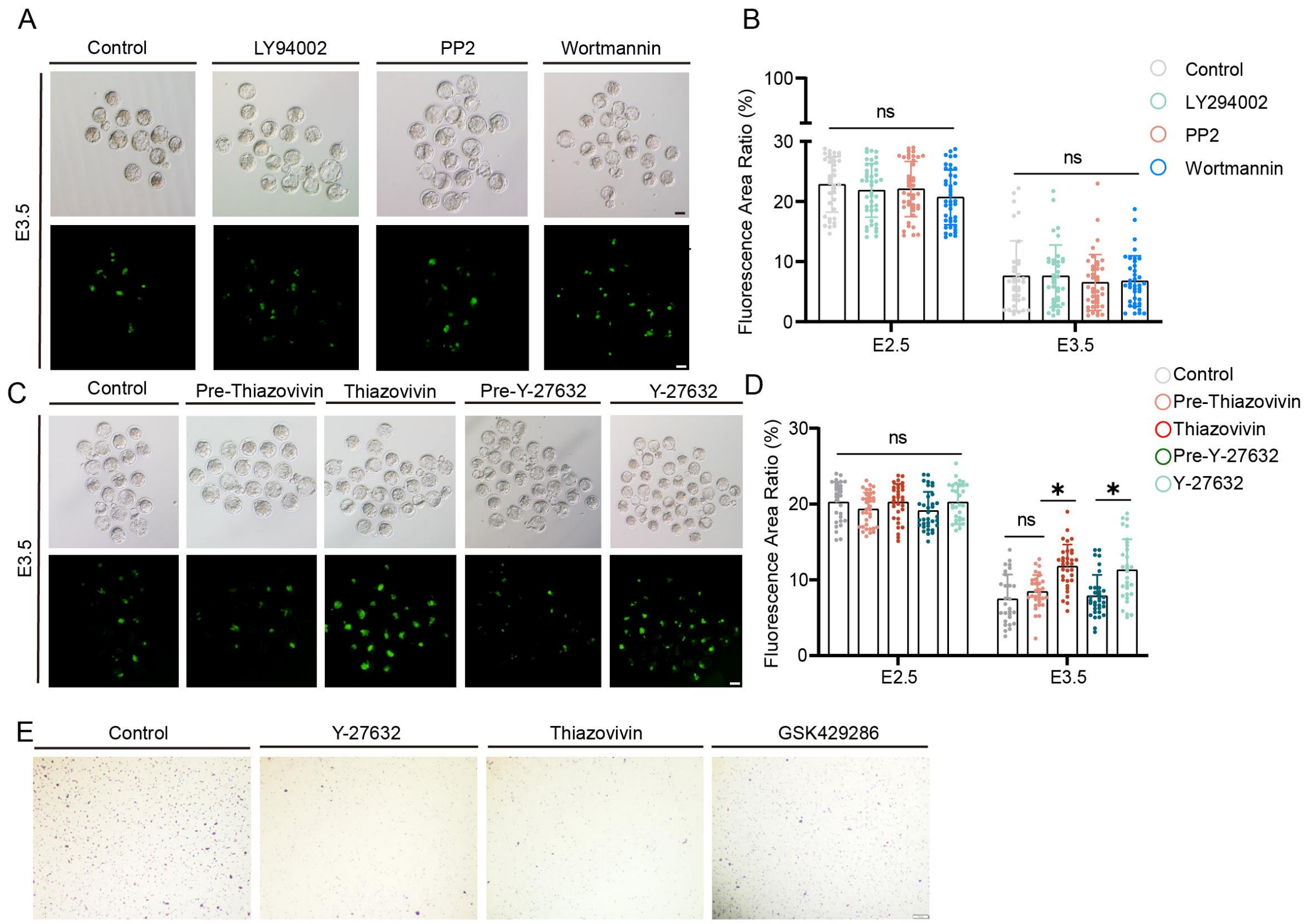

Figure S3. Pharmacological Screening and Treatment Strategies Comparison (Related to Figure 3)

(A) Pharmacological screening for phagocytosis inhibition. Representative fluorescence images of E3.5 embryos treated with PI3K inhibitors (Wortmannin, LY294002) and Src family kinase inhibitor (PP2). Scale bar = 100 μm

(B) Statistics of cell retention. Fluorescence area ratio (%) at E2.5 and E3.5 stages under different phagocytosis inhibitors, showing limited rescue effects compared to control. Statistical analysis of cell retention under various phagocytosis inhibitors. Data are presented as mean ± SD. ns, not significant, determined by one-way ANOVA followed by Tukey's post-hoc test. *n*=36 for Control, 41 for Wortmannin, 42 for LY294002, and 40 for PP2 (*N*=3 independent experiments).

(C) Comparison of pre-treatment vs. co-culture treatment. Representative images showing the effect of pre-incubating donor TSCs with ROCK inhibitors (Pre-Thiazovivin,

Pre-Y-27632) compared to active treatment during embryo chimerism.

(D) Quantitative statistics of treatment timing. Statistics of fluorescence area ratio at E2.5 and E3.5. Data are presented as mean ± SD. **P < 0.01, determined by one-way ANOVA followed by Tukey’s post-hoc test. *n*=32 for Control, 34 for Pre-Thiazovivin, 32 for Thiazovivin, 35 for Pre-Y-27632, and 31 for Y-27632 (*N*=3 independent experiments).

(E) Transwell migration assay. Quantification and representative images showing that ROCK inhibition impairs the migratory capability of TSCs. Scale bar =50 m

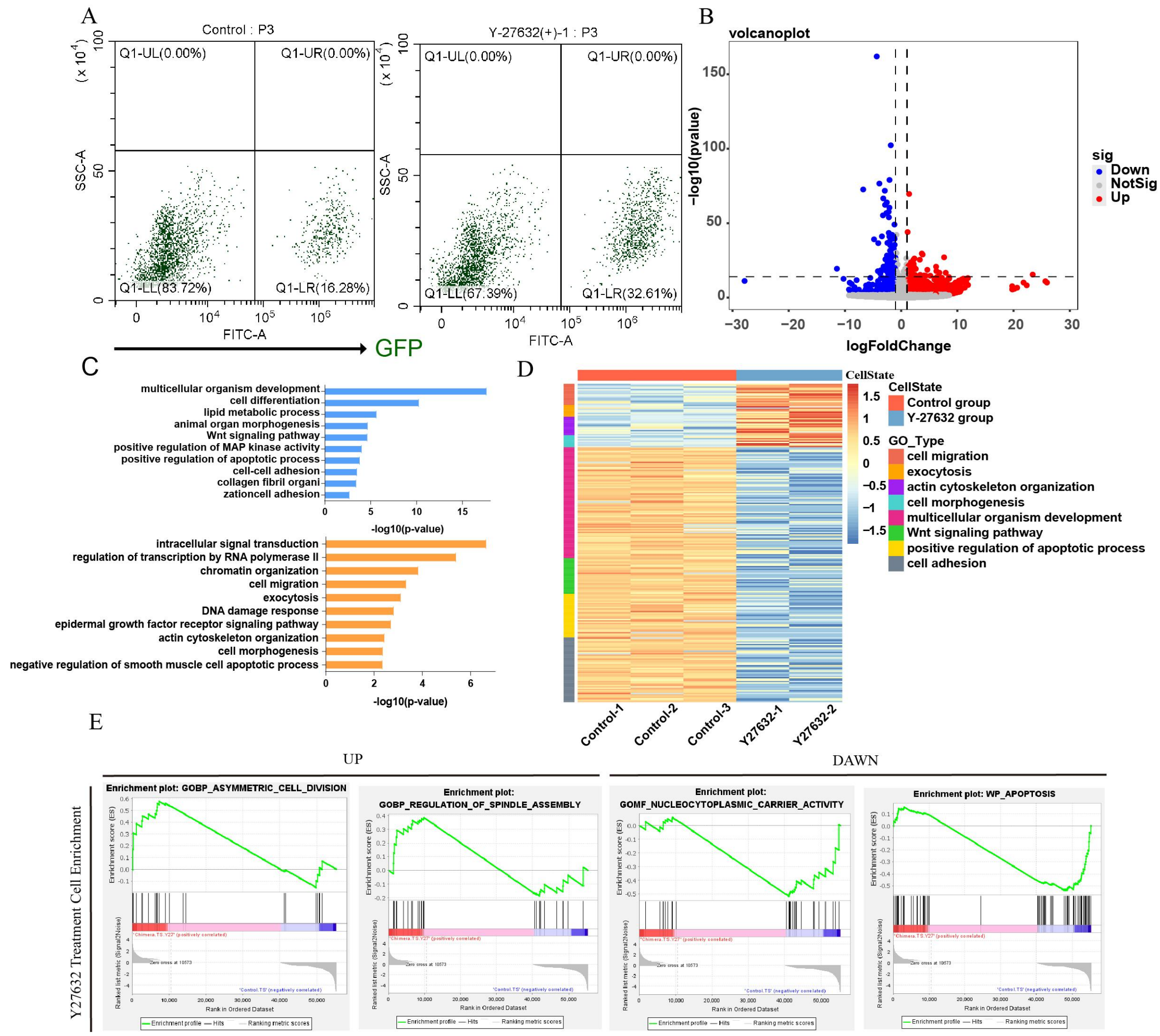

Figure S4. Transcriptomic Profiling and Long-term Flow Cytometry Validation. (Related to Figure 4)

(A) Flow cytometry (FACS) analysis of E12.5 placentas. Representative FACS plots showing the proportion of GFP-positive donor cells in the Y-27632-treated group versus the control group.

(B) Volcano plot of differentially expressed genes (DEGs). Comparison of gene expression between Y-27632-treated and control morula-stage TSCs ($|log2FC| > 1$, FDR < 0.05).

(C) Gene Ontology (GO) enrichment analysis. Functional annotation of DEGs highlighting pathways involved in actin cytoskeleton organization, cell migration, and

positive regulation of the apoptotic process.

(D) Cluster heatmap of functional genes. Expression profiles of genes associated with cytoskeletal remodeling, cell adhesion, and exocytosis in control versus Y-27632 treated groups.

(E) Gene Set Enrichment Analysis (GSEA). Enrichment plots for asymmetric cell division, regulation of spindle assembly, and apoptosis pathways following ROCK inhibition
