# Supplementary material for: ROCK inhibition suppresses host blastomere-mediated elimination and enhances trophoblast stem cell contribution in mouse chimeras
Source: Stem Cell Reports. 2026 Oct 1;21(10):103111. doi: 10.1016/j.stemcr.2026.103111 (PMC13681403; doi:10.1016/j.stemcr.2026.103111)
Supplement: Supplementary file 2 [file mmc5.pdf]

STEM CELL REPORTS
Report
OPEN ACCESS

ROCK inhibition suppresses host blastomere-mediated elimination and enhances trophoblast stem cell contribution in mouse chimeras

Baoxing Dong,[1,2,3] Kuisheng Liu,[1,3] Chenxiang Xi,[1,3] Sheng Wang,[1,3] Yifan Sheng,[1] Jiani Xiang,[1] Jiayu Chen,[1] Yingdong Liu,[1] Dandan Bai,[1] Shanyao Liu,[1] Xia Tu,[1] Xiaochen Kou,[1] Yanhong Zhao,[1] Jiqing Yin,[1] Hong Wang,[1] Binya Liu,[1,*] Changle Ma,[2,*] Shaorong Gao,[1,4,*] and Wenqiang Liu[1,*]
[1]Shanghai Key Laboratory of Maternal Fetal Medicine, Clinical and Translational Research Center of Shanghai First Maternity and Infant Hospital, Shanghai Institute of Maternal-Fetal Medicine and Gynecologic Oncology, Frontier Science Center for Stem Cell Research, School of Life Sciences and Technology, Tongji University, Shanghai 200092, China
[2]College of Life Sciences Technology, Shandong Normal University, Shandong 250358, China
[3]These authors contributed equally
[4]Lead contact
*Correspondence: liu.binya@tongji.edu.cn (B.L.), machangle@sdnu.edu.cn (C.M.), gaoshaorong@tongji.edu.cn (S.G.), liuwenqiang@tongji.edu.cn (W.L.)
https://doi.org/10.1016/j.stemcr.2026.103111

SUMMARY

Trophoblast stem cells (TSCs) play a crucial role in modeling the complex dynamics of maternal-fetal morphogenesis and placental development. However, their utility is hindered by low contribution rates in embryonic chimeras. Here, we reveal that during the 8-cell mouse embryo aggregation, host blastomeres selectively eliminate donor TSCs through an actin-dependent, phagocytic-like process and degrade them via lysosomal pathways, acting as a developmental quality-control mechanism. Notably, modulating cytoskeletal dynamics via Rho/ROCK pathway inhibition markedly suppresses this host-mediated clearance, enhancing donor TSC retention and contribution to extraembryonic tissues in pre- and post-implantation chimeras without compromising embryonic viability. Overall, our findings identify host blastomere-mediated elimination as an early barrier to TSC chimerism and establish transient ROCK inhibition as a strategy for improving TSC contribution during placental development.

INTRODUCTION

Trophoblast stem cells (TSCs) are indispensable for modeling placental development and pregnancy-related disorders (Tanaka et al., 1998). Establishment of embryonic chimeras represents the gold standard for assessing the developmental potential of diverse stem cell types. Compared with blastocyst chimeras, 8-cell stage injection is particularly advantageous as it effectively recapitulates the microenvironment required to evaluate extraembryonic lineage capacity (Stepien et al., 2021; Poueymirou et al., 2007). Specifically, generating 8-cell embryo chimeras using TSCs serves as a powerful tool to dissect the fate decisions of the trophoblast lineage and gain deeper insights into placenta-associated diseases (Huang et al., 2008).

However, the practical utility of TSCs is limited by their significantly lower chimeric efficiency relative to embryonic stem cells (ESCs) (Tanaka et al., 1998; Poueymirou et al., 2007). The cellular barriers underlying this discrepancy remain poorly understood (Huang et al., 2008). Here, we reveal that host blastomeres actively clear exogenous TSCs via a cellular internalization process; while referencing the epithelial phagocytosis observed in early embryos (Hoijman et al., 2021), this elimination machinery operates through a non-canonical, lineage-specific pathway. We propose this selective elimination as a key barrier to successful TSC integration. Notably, we demonstrate that targeting the Rho/ROCK axis, a pathway implicated in phagocytic regulation (Mao and Finnemann, 2015), blocks these internalization events and improves the contribution potential of TSCs to extraembryonic tissues in mouse chimeras.

RESULTS

Rapid clearance of chimeric TSCs in preimplantation embryos restricts their developmental contribution

To evaluate the lineage contribution potential of TSCs and ESCs, we utilized an 8-cell embryo injection platform (Figure 1A), an established system for exogenous cell integration (Tokunaga and Tsunoda, 1992). We established GFP-labeled ESC (González et al., 2010) and TSC (Tanaka et al., 1998) lines derived from transgenic mice expressing green fluorescent protein. Immunofluorescence (IF) and quantitative PCR (qPCR) confirmed that TSCs exhibited high levels of expression of CDX2 and TFAP2C, whereas ESCs showed robust OCT4 and NANOG expression (Figures S1A and S1B).

Following microinjection into 8-cell embryos, ESC-integrated chimeras achieved over 70% chimeric contribution in pre-implantation and post-implantation embryos (Figure 1B), consistent with prior reports (Poueymirou et al., 2007). In contrast, TSCs displayed significantly lower

© 2026 The Author(s).
Published by Elsevier Inc. on behalf of International Society for Stem Cell Research.
This is an open access article under the CC BY license (http://creativecommons.org/licenses/by/4.0/).

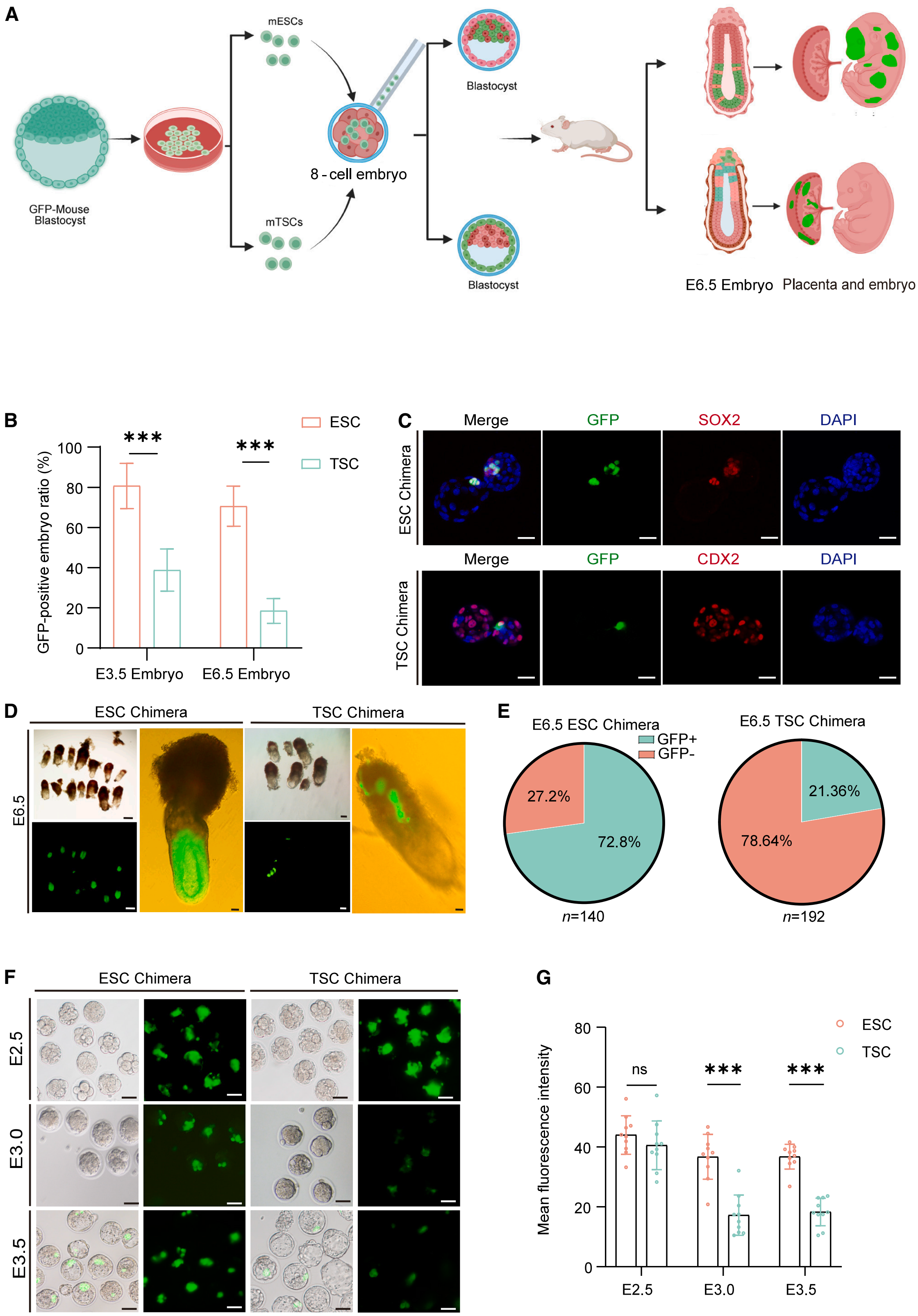

(legend on next page)

contribution rates at the blastocyst stage (Figures 1B and 1C). Post-implantation, the chimeric efficiency of TSCs in the extraembryonic lineage was further reduced while ESCs remained robust (Figures 1D and 1E; Figure S1C). Time-course analysis revealed that elimination of donor TSCs occurred precipitously between E2.5 and E3.0 and stabilized at a minimal level by E3.5 (Figures 1F and 1G). Collectively, these findings suggest that the low contribution potential of TSCs may result, at least in part, from active clearance during preimplantation embryo development.

Selective elimination of donor TSCs by host blastomeres via non-apoptotic phagocytosis

To investigate the rapid loss of TSCs, we injected GFP-labeled ESCs, primitive endoderm stem cells (PrESCs) (Ohinata et al., 2022), and TSCs into 8-cell stage mouse embryos. PrESCs were utilized as an extraembryonic lineage control to verify the specificity of TSC elimination. Dissociation analysis at the morula stage revealed that approximately 80% of ESCs and 70% of PrESCs persisted, whereas only 20% of TSCs remained (Figures 2A–2C). This lineage-specific disparity suggests selective elimination of TSCs by host blastomeres. Crucially, within 4 h post-injection, we observed TSC fluorescence colocalizing with host blastomere cytoplasm, indicating internalization (Figures 2D and 2E). Time-lapse imaging and confocal microscopy showed that TSCs are engulfed via plasma membrane invagination, a process morphologically distinct from classical pseudopod-mediated phagocytosis (Figures 2F and 2G; Figure S2A and Video S1). Concurrently, we quantified these internalization events during the initial two hours post-injection, further demonstrating the rapid and consistent nature of this elimination process (Figure 2H). Subsequent LysoTracker red labeling indicated lysosomal-associated acidic compartment involvement, suggesting acidic degradation of engulfed TSCs (Figure 2I). Collectively, these morphological and fluorescence observations indicate that host blastomeres actively eliminate donor TSCs through a phagocytic-like pathway.

We next investigated whether this elimination is a general property of the trophoblast lineage. Unlike ESCs and PrESCs (both from C57BL/6 × 129S1), multiple TSC lines exhibited uniform internalization behavior regardless of their developmental origin (Figures S2C–S2H). We utilized C57BL/6 × 129S1 TSCs, ITS, and E65TS cells, which were established from E3.5 ICR blastocysts and E6.5 postimplantation ICR embryos to represent pre- and postimplantation trophoblast lineages (Seong and Rivron, 2023; Kubaczka et al., 2014). These results confirm that selective clearance is a fundamental characteristic inherent to TSC identity rather than a cell-line-specific artifact.

To determine whether donor cell apoptosis triggers this host-mediated internalization, we first assessed endogenous cell death markers. No significant apoptosis was detected in injected ESCs, PrESCs, or TSCs during the initial period between microinjection and the onset of internalization (Figure S2B). To further test if the host phagocytic machinery is sensitive to the apoptotic status of donor cells, we exogenously induced early apoptosis in TSCs using H_2O_2 (Guo et al., 2010). Inducing early apoptosis in TSCs via H_2O_2 did not increase phagocytosis rates (Figure 2J). Furthermore, while survival-promoting agents, including the pan-caspase inhibitor Z-VAD-FMK (Slee et al., 1996), IGF-1 (Lighten et al., 1998), and resveratrol (Zhu et al., 2011) enhanced overall donor cell viability, they failed to prevent TSC clearance (Figures 2K and 2L).

Taken together, these results indicate that host blastomeres can eliminate exogenous TSCs through a specialized internalization process and subsequent degradation within acidic compartments.

Inhibiting cytoskeletal motility significantly reduces blastomere phagocytosis of TSCs

To dissect the underlying mechanisms of blastomere-mediated internalization, we performed a pharmacological

Figure 1. Selective elimination of TSCs in pre-implantation and post-implantation embryos

(A) Schematic workflow of chimeric embryo production using the 8-cell microinjection method.

(B) Statistical analysis of contribution rate of ESCs and TSCs to chimeras at E3.5 and E6.5 stages. Comparisons were performed separately at each developmental stage. Data are presented as mean ± SD. ***$p < 0.001$, determined by unpaired two-tailed Student's t test. For E3.5 chimeras, $n = 87$ embryos (ESCs) and 103 embryos (TSCs) from $N = 4$ independent experiments. For E6.5 chimeras, $n = 140$ embryos (ESC, $N = 3$ independent experiments) and 192 embryos (TSC, $N = 6$ independent experiments).

(C) Representative immunofluorescence (IF) images of E3.5 chimeric blastocysts showing the integration of donor ESCs ($SOX2^+$) or TSCs ($CDX2^+$). Scale bars, 20 μm.

(D) Bright-field and fluorescence images of chimeric embryos at the E6.5 stage. Scale bars, 1 mm.

(E) Pie charts showing the contribution rate of ESC and TSC to chimeras in E6.5 embryos. GFP^+ (green) indicates successful chimerism; GFP^- (salmon) indicates no donor cell contribution.

(F) Time-lapse sequences showing the dynamics of chimeric cells from E2.5 to E3.5. Scale bars, 100 μm.

(G) Quantitative analysis of mean fluorescence intensity for GFP-labeled TSCs across pre-implantation developmental stages. Data are presented as mean ± SD. ***$p < 0.001$, determined by unpaired Student's t test. Data were quantified using ImageJ from $n = 10$ randomly selected embryos per stage.

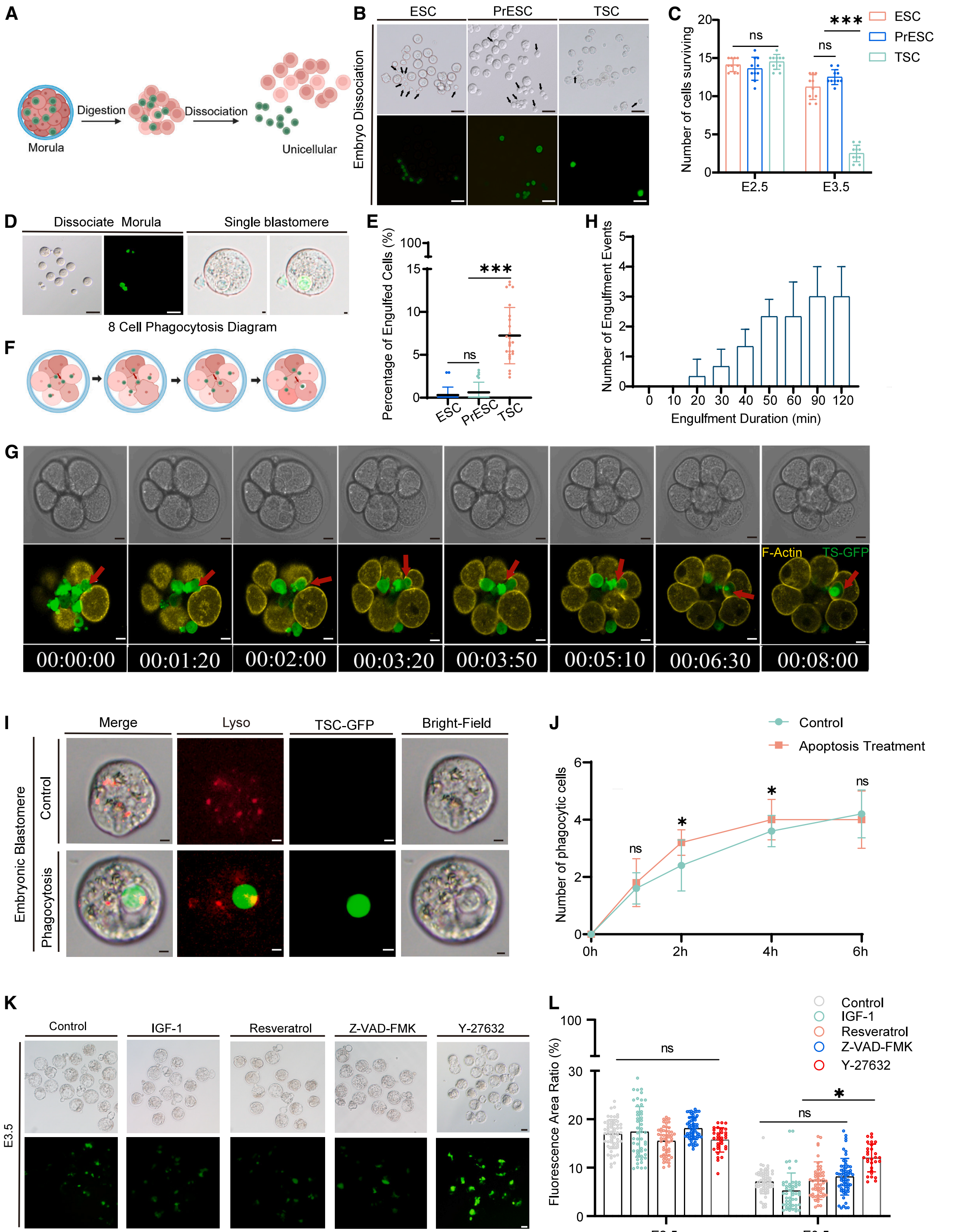

(legend on next page)

screen targeting canonical phagocytic and signaling pathways. Wortmannin, LY294002 (Araki et al., 1996) (PI3K inhibitors to block phagosome maturation) and PP2 (Fitzer-Attas et al., 2000) (an Src family kinase inhibitor) failed to significantly reduce TSC internalization (Figures S3A and S3B), suggesting that TSC engulfment bypasses classical phagocytic signaling.

Given the prominent cytoskeletal remodeling observed, we tested the role of ACTIN in this process using structural probes. ACTIN disruptors (Brodin and Shupliakov, 2025) (cytochalasin D and latrunculin A) effectively abolished TSC internalization in a dose-dependent manner (Figures 3A and 3B), although they caused morula-stage developmental arrest. Consistent with the requirement for active cytoskeletal remodulation, high-magnification imaging drastically diminished clearance of donor TSCs, enabling their stable persistence and integration within host blastomeres (Figures 3C and 3D).

We next examined small molecules regulating cytoskeletal dynamics. ROCK inhibitors block the kinase activity of ROCK by competitively binding to its ATP-binding pocket to suppress downstream cytoskeletal rearrangement (Riento and Ridley, 2003; Thumkeo et al., 2013). Targeting the Rho/ROCK pathway using Y-27632, Thiazovivin, or GSK429286 (Pan et al., 2013) led to a marked reduction in TSC internalization, while embryonic viability remained unaffected (Figures 3E and 3F) (Watanabe et al., 2007). Unlike the survival-promoting agents and pan-caspase inhibitors tested earlier, which failed to yield comparable outcomes (Figures 2K and 2L), disruption of the Rho/ROCK axis appears to alleviate cell clearance. These collective observations imply that TSC elimination may be modulated by mechanical factors rather than being solely driven by the apoptotic status of donor cells.

Real-time time-lapse imaging captured the dynamic suppression of this barrier: in control embryos, host blastomeres rapidly formed actin-rich phagocytic cups to internalize TSCs (May and Machesky, 2001) (Video S2). Conversely, ROCK inhibition significantly impeded the formation of these cups, allowing donor cells to remain stably integrated at the cell-cell interface (Video S3). ROCK inhibitors significantly reduced the number of membrane protrusions at the host-donor interface (Figures 3G and 3H) and impaired the migration capability of TSCs (Figures 3I and S3E). These findings suggest that ROCK inhibitor reduces blastomere phagocytosis of TSCs by inhibiting cytoskeletal motility.

ROCK inhibition enhances the chimerization potential of TSCs in post-implantation embryos

To evaluate the long-term impact of overcoming the initial phagocytic barrier, we examined the chimerization potential of TSCs following ROCK inhibition across multiple developmental stages. In E3.5 blastocysts, Y-27632 and GSK429286 effectively promoting specific integration into the trophectoderm (TE) (Figures 4A and 4B). The stability of this integration was further validated in post-implantation embryos. In E6.5 embryos, Y-27632-treated chimeras exhibited robust

Figure 2. Host blastomeres eliminate donor TSCs via cell-type-specific phagocytosis

(A) Schematic of the dissociation protocol used to assess donor cell viability in morula-stage chimeras.

(B) Representative images of donor cells (ESC, PrESC, or TSC) recovered after embryo dissociation. Black arrows indicate integrated GFP+ cells. Scale bars, 20 μm.

(C) Statistical analysis of the number of surviving versus lost donor cells at the morula stage (E2.5 and E3.5). Data are presented as mean ± SD.***$p < 0.001$, determined by an unpaired two-tailed Student's t test. $n = 10$ randomly selected embryos per cell line.

(D) High-magnification images showing the cytoplasmic colocalization of a donor TSC (GFP$^+$) within a host blastomere, indicating internalization. Scale bars, 5 μm.

(E) Percentage of engulfed donor cells in ESC, PrESC, and TSC chimeric embryos. Data are presented as mean ± SD, ***$p < 0.001$, determined by unpaired Student's t test. $n = 25$ randomly selected embryos per cell line.

(F) Schematic diagram of the active phagocytosis mechanism in 8-cell stage embryos.

(G) Time-lapse confocal imaging of TSC internalization. F-actin (yellow) reorganizes to form a phagocytic cup around the donor TSC (green). Red arrows indicate donor cells undergoing internalization by host blastomeres. Scale bars, 10 μm.

(H) Frequency distribution and duration of TSC engulfment events. $n = 39$ engulfment events recorded from 20 embryos. Data were analyzed using ImageJ and GraphPad Prism.

(I) Verification of lysosome involvement using LysoTracker red, showing rapid accumulation around internalized TSCs. Scale bars, 10 μm.

(J) Comparison of TSC engulfment frequency over time between control and apoptosis-induced (H_2O_2-treated) groups. Data are presented as mean ± SD. *$p < 0.05$, determined by unpaired Student's t test. For Control, $n = 28$ embryos $N = 3$ independent experiments and for apoptosis-induced groups, $n = 27$ embryos $N = 3$ independent experiments.

(K) Representative images of E3.5 TSC chimeric embryos under various treatments (Control, IGF-1, Resveratrol, Z-VAD-FMK, and Y-27632). Scale bars, 50 μm.

(L) Statistics of fluorescence area ratio (%) at E2.5 and E3.5 stages across various apoptosis inhibitors treatment groups. Data are presented as mean ± SD. *$p < 0.05$, was assessed by one-way ANOVA followed by Tukey's post-hoc test. $n = 57$ for control, 50 embryos for IGF-1, 55 embryos for resveratrol, 61 embryos for Z-VAD-FMK, and 31 embryos for Y-27632.

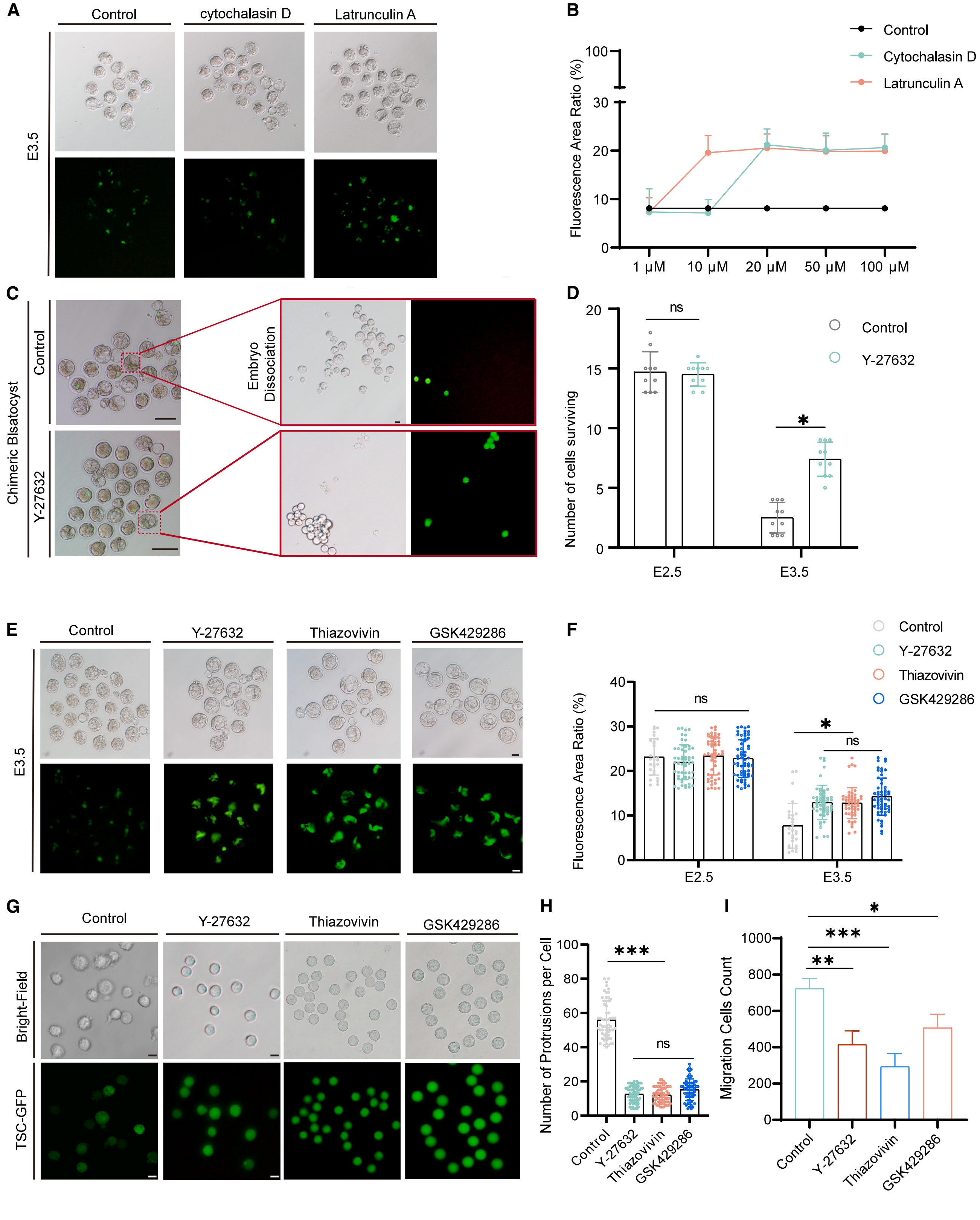

(legend on next page)

TFAP2C$^+$ and GFP$^+$ TSC contribution within the extraembryonic ectoderm (ExE) and ectoplacental cone (EPC), whereas controls showed only sparse remnants (Figures 4C and 4D). To examine the long-term persistence of donor TSC contribution during placental development, we examined E12.5 conceptuses using a dual-tracking strategy. Whole-mount fluorescence imaging of intact placentas revealed a notably higher density of GFP-positive cellular foci in the Y-27632-treated group compared to the control (Figure 4E). Longitudinal cross-section immunofluorescence further demonstrated a significantly increased donor-occupied area ratio within the placental layers upon ROCK inhibition (Figures 4F and 4G). Consistently, flow cytometry (FACS) analysis of dissociated placental cells confirmed a significant upregulation in the absolute proportions of GFP-positive donor cells in the Y-27632-supplemented group (Figure S4A). To systematically explore the molecular changes driving this optimization, transcriptomic profiling comparison between morula-stage embryos and TS donor cells revealed that transient ROCK inhibition differentially modulated regulatory pathways predominantly enriched in cell migration, actin cytoskeleton organization, and exocytosis (Figures 4H–4J; Figure S4B–S4E).

Collectively, these findings revealed that pharmacological inhibition of ROCK signaling reduces early donor-cell elimination and improves long-term TSC contribution during placental morphogenesis.

DISCUSSION

While apoptotic cell clearance is a classical mechanism for maintaining developmental homeostasis (Mirkes, 2008), our study shows that TSC elimination in 8-cell embryos is functionally decoupled from the apoptotic program. The failure of anti-apoptotic treatments—including Z-VAD-FMK, IGF-1, and resveratrol (Zhu et al., 2011)—to rescue TSC contribution to chimeras confirms that donor cell viability is not the primary determinant for host recognition. Based on the selective nature of TSC elimination, we propose a stepwise model involving putative donor-cell recognition, actin-dependent internalization, and subsequent lysosomal degradation (Hoijman et al., 2021; Fitzer-Attas et al., 2000). This process is consistent with a specialized active quality control mechanism that may operate in early embryos.

Although transcriptomic data indicated shifts in apoptotic signatures following ROCK inhibition, the functional failure of survival factors suggests these changes are secondary effects. These data suggest that the improved chimerization potential of TSCs is primarily associated with disruption of the mechanical internalization machinery. Specifically, while global actin disruptors (cytochalasin D and latrunculin A) abolished internalization at the cost of concomitant developmental arrest, targeted inhibition of the Rho/ROCK axis effectively blocked phagocytic cup formation without compromising embryonic viability. These observations are consistent with the notion that engulfment is primarily driven by dynamic, highly localized actin remodeling.

The contribution of TSCs to chimerism is constrained by the developmental disparity between donor cells and the host environment. When injected into blastocysts or 8-cell stage embryos, TSCs face a developmental mismatch, which likely marks them as distinct cellular population and hampers their contribution to chimerism.

Figure 3. Regulation of donor cell internalization by actomyosin dynamics and the Rho/ROCK axis
(A) Fluorescence images of E3.5 chimeric embryos treated with actin disruptors (Cyto D, Lat A). Scale bars, 50 μm.
(B) Dose-response curves showing the fluorescence area ratio (%) following treatment with varying concentrations of cytoskeletal inhibitors. Data are presented as mean ± SD. *$p < 0.05$, determined by one-way ANOVA followed by Tukey's post-hoc test. $n = 46$ embryos for control, 55 embryos for cytochalasin D, 51 embryos for latrunculin A.
(C) Representative images of dissociated chimeric blastocysts following Y-27632 treatment compared to control. Scale bars, 150 μm.
(D) Quantification of surviving TSCs in the dissociated chimeric blastocysts treated with Y-27632 treatment compared to control. Data are presented as mean ± SD.*$p < 0.05$, was determined by unpaired Student's t test. $n = 10$ randomly selected embryos.
(E) Images of TSC chimeras treated with different ROCK inhibitors (control, Y-27632, Thiazovivin, and GSK429286). Scale bars, 20 μm.
(F) Statistical analysis of the fluorescence area ratio (%) at E3.5 following treatment with different ROCK inhibitors. Fluorescence area was quantified via ImageJ. Data are presented as mean ± SD. *$p < 0.05$, determined by one-way ANOVA followed by Tukey's post-hoc test. $n = 28$ embryos for control, 54 embryos for Y-27632, 52 embryos for Thiazovivin, and 58 embryos for GSK429286.
(G) Representative bright-field and fluorescence images of membrane protrusions in donor TSCs treated with ROCK inhibitors. Scale bars, 10 μm.
(H) ImageJ-based quantification of membrane protrusions per cell across different treatment groups. Data are presented as mean ± SD. ***$p < 0.001$, determined by one-way ANOVA followed by Tukey's post-hoc test. $n = 59$ embryos for control, 64 embryos for Y-27632, 36 embryos for Thiazovivin, and 47 embryos for GSK429286.
(I) Transwell migration assay showing the quantification of migrated donor TSCs following Y-27632 treatment *in vitro*. Data are presented as mean ± SD; *$p < 0.05$, **$p < 0.01$, ***$p < 0.001$, determined by unpaired two-tailed Student's t test. $n = 5$ randomly selected microscopic fields per insert from $N = 3$ independent experiments.

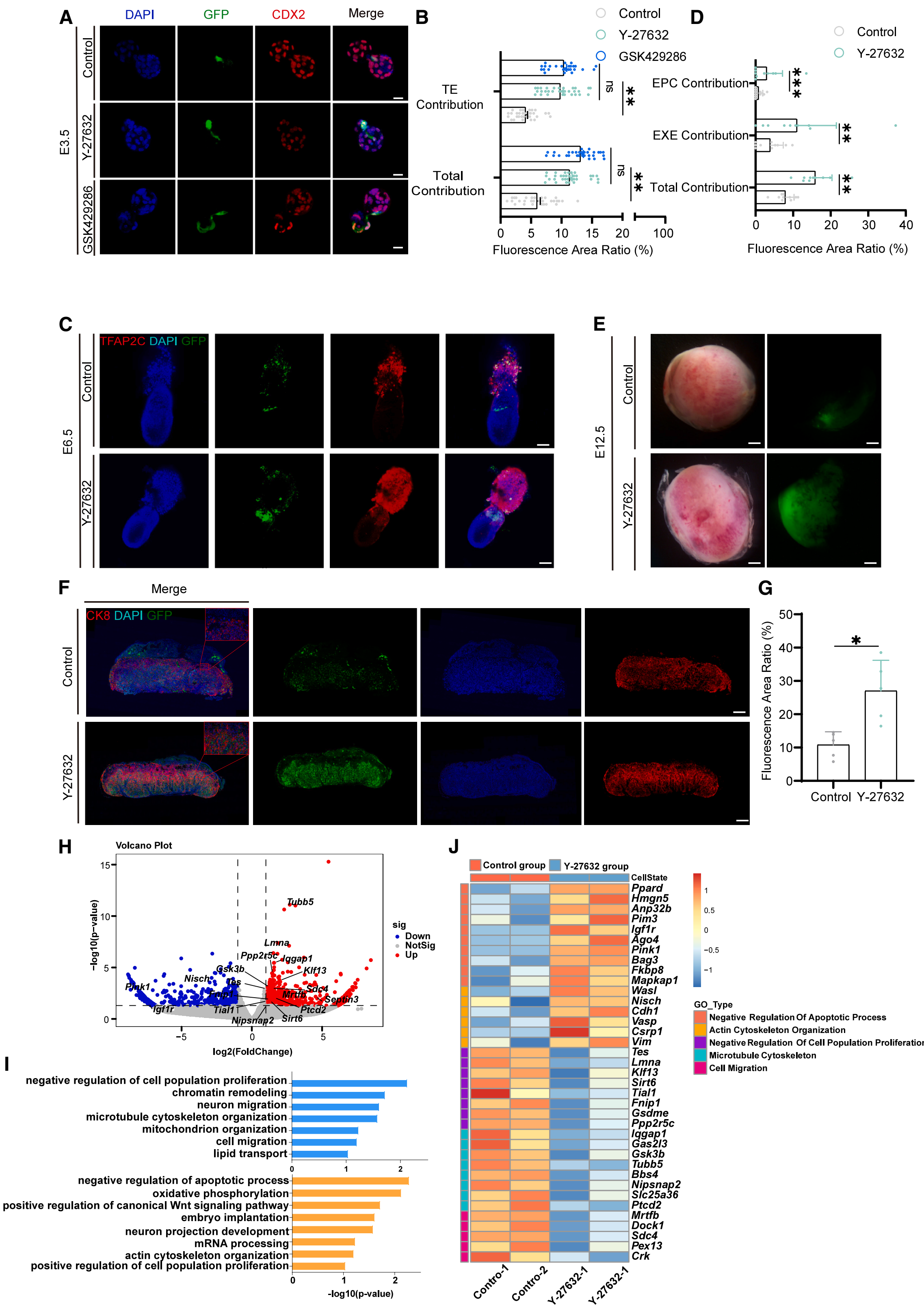

(legend on next page)

Transient ROCK inhibition allows these developmentally advanced cells to bypass the initial compatibility barrier and pre-occupy the extraembryonic niche before host lineage commitment is finalized.

Our long-term *in vivo* validation underscores the practical utility of this strategy. Beyond early integration, FACS and histological analyses confirmed that ROCK inhibition leads to sustained donor cell contribution within E12.5 placentas. The successful colonization suggests that bypassing the 8-cell barrier is a viable strategy to enhance the developmental potential of TSCs *in vivo* (Seong et al., 2022; Harrison et al., 2018; Wang et al., 2023b). This approach may also mitigate mechanical elimination barriers observed in interspecies chimeras (Harrison et al., 2018; Wang et al., 2023b; Zheng et al., 2021).

Despite these advancements, the specific cell-surface receptors mediating initial recognition remain unidentified. Our findings suggest that selective ligand-receptor interactions trigger host actin recruitment. Future research focusing on "eat-me" or "don't-eat-me" signals (Wang et al., 2023a) and the extracellular matrix (ECM) molecules that modulate them (Wang et al., 2023b) will be essential to fully elucidate embryonic developmental quality control (Mirkes, 2008; Seong et al., 2022; Di Gregorio et al., 2016).

RESOURCE AVAILABILITY

Lead contact

Further information and requests for resources and reagents should be directed to the lead contact, Shaorong Gao (gaoshaorong@tongji.edu.cn).

Materials availability

All the materials generated and used in this study will be available upon reasonable request.

Data and code availability

- This paper does not report original code. RNA sequencing data have been deposited in the Genome Sequence Archive (GSA) repository with accession number GSA: PRJCA0 67470.
- The RNA-seq data generated in this study have been deposited in the Genome Sequence Archive (GSA) under accession number GSA: PRJCA067470. This paper does not report original code. Any additional information required to reanalyze the data reported in this paper is available from the lead contact upon reasonable request.

ACKNOWLEDGMENTS

This work was supported by the National Natural Science Foundation of China (32488101, 32330030, 32570948, 32270909, 32400677, 324B2021, and 31820103009), Fundamental and Interdisciplinary Disciplines Breakthrough Plan of the Ministry of Education of China (no. JYB2025XDXM508), the key project of the Science and Technology of Shanghai Municipality (21JC1405500), Shanghai Pilot Program for Basic Research, Shanghai "Medical Academy Rising Star" Young Medical Talents Program supported by Shanghai Municipal Health, "Talent Reservoir" Sailing Program supported by Shanghai First Maternity and Infant Hospital, Shanghai Post-doctoral Excellence Program (2023616), China Postdoctoral Science Foundation (2023M732660), Postdoctoral Fellowship Program of CPSF (GZB20230523), the Fundamental Research Funds for the Central Universities (22120240435), Shenzhen Medical Research Fund (B2402013), and Peak Disciplines (Type IV) of Institutions of Higher Learning in Shanghai.

AUTHOR CONTRIBUTIONS

W.L., C.M., B.L., and S.G. conceived and designed the experiments. B.D., K.L., and C.X. performed most of the experiments. S.W. performed the computational analysis. Y.S., J.X., J.C., Y.L.,

Figure 4. Enhancement of TSC contribution potential via ROCK inhibition

(A) IF staining of E3.5 chimeric embryos (DAPI, GFP, and CDX2) under ROCK inhibition (Y-27632 and GSK429286). Scale bars, 20 μm.

(B) Statistics comparing TE-specific contribution ratios versus total contribution ratios. Data are presented as mean ± SD. $^{**}p < 0.01$, determined by one-way ANOVA followed by Tukey's multiple-comparisons test. $n = 41$ embryos for control, 32 embryos for Y-27632, and 47 embryos for GSK429286.

(C) Fluorescence images of E6.5 chimeric embryos, with dashed lines outlining the ExE, EPC, and Epi regions. Scale bars, 1 mm.

(D) Quantitative analysis of donor cell contribution to extraembryonic regions (ExE, EPC) and the embryo proper (Epi) at E6.5. Data are presented as mean ± SD. $^{*}p < 0.05$, $^{**}p < 0.01$, determined by two-way ANOVA. $n = 10$ embryos per group.

(E) Whole-mount fluorescence images of E12.5 placentas in control and Y-27632 groups. Scale bars, 1 mm.

(F) IF staining of placental longitudinal cross-sections (CK8, DAPI, and GFP) showing the distribution of donor cells across placental layers. Placentas are oriented with the maternal decidua at the top. Scale bars, 50 μm.

(G) Morphometric quantification of the GFP-positive fluorescence area ratio relative to the total placental area. Stained longitudinal sections were analyzed via ImageJ to determine the percentage of donor-occupied area over the entire placental organ. Data are presented as mean ± SD. $^{*}p < 0.05$, determined by an unpaired two-tailed Student's t test. $n = 5$ placentas per group.

(H) Volcano plot showing differentially expressed genes (DEGs) in morula embryos treated with Y-27632.

(I) Gene Ontology (GO) enrichment analysis of DEGs, highlighting the negative regulation of apoptosis and actin cytoskeleton organization.

(J) Cluster heatmap showing the hierarchical clustering of representative DEGs associated with actin cytoskeleton organization, cell motility, and growth regulation profiles.

D.B., S.L., X.T., X.K., Y.Z., J.Y., and H.W. assisted with the experiments. D.B., J.S., W.L., C.J., and S.G. wrote the manuscript.

DECLARATION OF INTERESTS

The authors declare no competing interests.

STAR★METHODS

Detailed methods are provided in the online version of this paper and include the following:

- KEY RESOURCES TABLE
- EXPERIMENTAL MODEL AND STUDY PARTICIPANT DETAILS
  - Animals
  - Stem cell lines
- METHOD DETAILS
  - Embryo collection and processing
  - Chimera embryo production
  - Cell dissociation
  - Transwell migration assay
  - RNA extraction and RT-qPCR
  - Immunofluorescence staining
  - Smart-seq2 library preparation and sequencing
  - Live-cell imaging and internalization-event tracking
  - LysoTracker labeling
  - Karyotype analysis
  - Pharmacological treatments and apoptosis induction
  - Processing of E12.5 placentas for FACS
  - Smart-seq2 analysis
  - Image analysis and quantification using ImageJ
- QUANTIFICATION AND STATISTICAL ANALYSIS

SUPPLEMENTAL INFORMATION

Supplemental information can be found online at https://doi.org/10.1016/j.stemcr.2026.103111.

Received: November 23, 2025
Revised: September 1, 2026
Accepted: September 2, 2026
Published: October 1, 2026

REFERENCES

Araki, N., Johnson, M.T., and Swanson, J.A. (1996). A role for phosphoinositide 3-kinase in the completion of macropinocytosis and phagocytosis by macrophages. J. Cell Biol. *135*, 1249–1260. https://doi.org/10.1083/jcb.135.5.1249.

Brodin, L., and Shupliakov, O. (2025). Actin cage for the synaptic vesicle liquid phase. Cell Rep. *44*, 116630. https://doi.org/10.1016/j.celrep.2025.116630.

Di Gregorio, A., Bowling, S., and Rodriguez, T.A. (2016). Cell competition and its role in the regulation of cell fitness from development to cancer. Dev. Cell *38*, 621–634. https://doi.org/10.1016/j.devcel.2016.08.012.

Dobin, A., Davis, C.A., Schlesinger, F., Drenkow, J., Zaleski, C., Jha, S., Batut, P., Chaisson, M., and Gingeras, T.R. (2013). STAR: ultrafast universal RNA-seq aligner. Bioinformatics *29*, 15–21. https://doi.org/10.1093/bioinformatics/bts635.

Fitzer-Attas, C.J., Lowry, M., Crowley, M.T., Finn, A.J., Meng, F., DeFranco, A.L., and Lowell, C.A. (2000). Fcgamma receptor-mediated phagocytosis in macrophages lacking the Src family tyrosine kinases Hck, Fgr, and Lyn. J. Exp. Med. *191*, 669–682. https://doi.org/10.1084/jem.191.4.669.

González, S., Ibáñez, E., and Santaló, J. (2010). Establishment of mouse embryonic stem cells from isolated blastomeres and whole embryos using three derivation methods. J. Assist. Reprod. Genet. *27*, 671–682. https://doi.org/10.1007/s10815-010-9473-9.

Guo, Y.L., Chakraborty, S., Rajan, S.S., Wang, R., and Huang, F. (2010). Effects of oxidative stress on mouse embryonic stem cell proliferation, apoptosis, senescence, and self-renewal. Stem Cell. Dev. *19*, 1321–1331. https://doi.org/10.1089/scd.2009.0313.

Harrison, S.E., Sozen, B., and Zernicka-Goetz, M. (2018). In vitro generation of mouse polarized embryo-like structures from embryonic and trophoblast stem cells. Nat. Protoc. *13*, 1586–1602. https://doi.org/10.1038/s41596-018-0005-x.

Hoijman, E., Häkkinen, H.M., Tolosa-Ramon, Q., Jiménez-Delgado, S., Wyatt, C., Miret-Cuesta, M., Irimia, M., Callan-Jones, A., Wieser, S., and Ruprecht, V. (2021). Cooperative epithelial phagocytosis enables error correction in the early embryo. Nature *590*, 618–623. https://doi.org/10.1038/s41586-021-03200-3.

Huang, J., Deng, K., Wu, H., Liu, Z., Chen, Z., Cao, S., Zhou, L., Ye, X., Keefe, D.L., and Liu, L. (2008). Efficient production of mice from embryonic stem cells injected into four- or eight-cell embryos by piezo micromanipulation. Stem Cell. *26*, 1883–1890. https://doi.org/10.1634/stemcells.2008-0164.

Kubaczka, C., Senner, C., Araúzo-Bravo, M.J., Sharma, N., Kuckenberg, P., Becker, A., Zimmer, A., Brüstle, O., Peitz, M., Hemberger, M., and Schorle, H. (2014). Derivation and maintenance of murine trophoblast stem cells under defined conditions. Stem Cell Rep. *2*, 232–242. https://doi.org/10.1016/j.stemcr.2013.12.013.

Krueger, F., James, F., Ewels, P., Afyounian, E., Weinstein, M., Schuster-Boeckler, B., and Hulselmans, G. (2021). FelixKrueger/TrimGalore: v0.6.7. Zenodo. https://doi.org/10.5281/zenodo.5127899.

Liao, Y., Smyth, G.K., and Shi, W. (2014). featureCounts: an efficient general pur-pose program for assigning sequence reads to genomic features. Bioinformatics *30*, 923–930. https://doi.org/10.1093/bioinformatics/btt656.

Lighten, A.D., Moore, G.E., Winston, R.M., and Hardy, K. (1998). Routine addition of human insulin-like growth factor-I ligand could benefit clinical in-vitro fertilization culture. Hum. Reprod. *13*, 3144–3150. https://doi.org/10.1093/humrep/13.11.3144.

Love, M.I., Huber, W., and Anders, S. (2014). Moderated estimation of fold change and dispersion for RNA-seq data with DESeq2. Genome Biol. *15*, 550. https://doi.org/10.1186/s13059-014-0550-8.

Mao, Y., and Finnemann, S.C. (2015). Regulation of phagocytosis by Rho GTPases. Small GTPases *6*, 89–99. https://doi.org/10.4161/21541248.2014.989785.

May, R.C., and Machesky, L.M. (2001). Phagocytosis and the actin cytoskeleton. J. Cell Sci. *114*, 1061–1077. https://doi.org/10.1242/jcs.114.6.1061.

Mirkes, P.E. (2008). Cell death in normal and abnormal development. Congenital. Anom. *48*, 7–17. https://doi.org/10.1111/j.1741-4520.2007.00175.x.

Ohinata, Y., Endo, T.A., Sugishita, H., Watanabe, T., Iizuka, Y., Kawamoto, Y., Saraya, A., Kumon, M., Koseki, Y., Kondo, T., et al. (2022). Establishment of mouse stem cells that can recapitulate the developmental potential of primitive endoderm. Science *375*, 574–578. https://doi.org/10.1126/science.aay3325.

Pan, P., Shen, M., Yu, H., Li, Y., Li, D., and Hou, T. (2013). Advances in the development of Rho-associated protein kinase (ROCK) inhibitors. Drug Discov. Today *18*, 1323–1333. https://doi.org/10.1016/j.drudis.2013.07.003.

Picelli, S., Faridani, O.R., Björklund, A.K., Winberg, G., Sagasser, S., and Sandberg, R. (2014). Full-length RNA-seq from single cells using Smart-seq2. Nat Protoc *9*. 171–81. https://doi.org/10.1038/nprot.2014.006.

Poueymirou, W.T., Auerbach, W., Frendewey, D., Hickey, J.F., Escaravage, J.M., Esau, L., Doré, A.T., Stevens, S., Adams, N.C., Dominguez, M.G., et al. (2007). F0 generation mice fully derived from gene-targeted embryonic stem cells allowing immediate phenotypic analyses. Nat. Biotechnol. *25*, 91–99. https://doi.org/10.1038/nbt1263.

Riento, K., and Ridley, A.J. (2003). Rocks: multifunctional kinases in cell behaviour. Nat. Rev. Mol. Cell Biol. *4*, 446–456. https://doi.org/10.1038/nrm1128.

Schindelin, J., Arganda-Carreras, I., Frise, E., Kaynig, V., Longair, M., Pietzsch, T., Preibisch, S., Rueden, C., Saalfeld, S., Schmid, B., et al. (2012). Fiji: an open-source platform for biological-image analysis. Nat. Methods *9*, 676–682. https://doi.org/10.1038/nmeth.2019.

Seong, J., and Rivron, N.C. (2023). Protocol for capturing trophectoderm stem cells reflecting the blastocyst stage. STAR Protoc. *4*, 102151. https://doi.org/10.1016/j.xpro.2023.102151.

Seong, J., Frias-Aldeguer, J., Holzmann, V., Kagawa, H., Sestini, G., Heidari Khoei, H., Scholte Op Reimer, Y., Kip, M., Pradhan, S.J., Verwegen, L., et al. (2022). Epiblast inducers capture mouse trophectoderm stem cells in vitro and pattern blastoids for implantation in utero. Cell Stem Cell *29*, 1102–1118.e8. https://doi.org/10.1016/j.stem.2022.06.002.

Slee, E.A., Zhu, H., Chow, S.C., MacFarlane, M., Nicholson, D.W., and Cohen, G.M. (1996). Benzyloxycarbonyl-Val-Ala-Asp (OMe) fluoromethylketone (Z-VAD.FMK) inhibits apoptosis by blocking the processing of CPP32. Biochem. J. *315*, 21–24. https://doi.org/10.1042/bj3150021.

Stepien, B.K., Vaid, S., Naumann, R., Holtz, A., and Huttner, W.B. (2021). Generation of interspecies mouse-rat chimeric embryos by embryonic stem (ES) cell microinjection. STAR Protoc. *2*, 100494. https://doi.org/10.1016/j.xpro.2021.100494.

Tanaka, S., Kunath, T., Hadjantonakis, A.K., Nagy, A., and Rossant, J. (1998). Promotion of trophoblast stem cell proliferation by FGF4. Science *282*, 2072–2075. https://doi.org/10.1126/science.282.5396.2072.

Thumkeo, D., Watanabe, S., and Narumiya, S. (2013). Physiological roles of Rho and Rho effectors in mammals. Eur. J. Cell Biol. *92*, 303–315. https://doi.org/10.1016/j.ejcb.2013.10.002.

Tokunaga, T., and Tsunoda, Y. (1992). Efficacious production of viable germ-line chimeras between embryonic stem (ES) cells and 8-cell stage embryos. Dev. Growth Differ. *34*, 561–566. https://doi.org/10.1111/j.1440-169X.1992.00561.x.

Wang, Y., Sun, J., Zhu, K., Wang, D., Zhao, X., Zhang, H., Wu, S., Wang, Y., and Wang, J. (2023a). Microglial aryl hydrocarbon receptor enhances phagocytic function via SYK and promotes remyelination in the cuprizone mouse model of demyelination. J. Neuroinflammation *20*, 83. https://doi.org/10.1186/s12974-023-02764-3.

Wei, Y., Zhang, E., Yu, L., Ci, B., Sakurai, M., Guo, L., Zhang, X., Lin, S., Takii, S., Liu, L., et al. (2023b). Dissecting embryonic and extraembryonic lineage crosstalk with stem cell co-culture. Cell *186*, 5859–5875.e24. https://doi.org/10.1016/j.cell.2023.11.008.

Watanabe, K., Ueno, M., Kamiya, D., Nishiyama, A., Matsumura, M., Wataya, T., Takahashi, J.B., Nishikawa, S., Nishikawa, S.i., Muguruma, K., and Sasai, Y. (2007). A ROCK inhibitor permits survival of dissociated human embryonic stem cells. Nat. Biotechnol. *25*, 681–686. https://doi.org/10.1038/nbt1310.

Zheng, C., Hu, Y., Sakurai, M., Pinzon-Arteaga, C.A., Li, J., Wei, Y., Okamura, D., Ravaux, B., Barlow, H.R., Yu, L., et al. (2021). Cell competition constitutes a barrier for interspecies chimerism. Nature *592*, 272–276. https://doi.org/10.1038/s41586-021-03273-0.

Zhu, X., Liu, Q., Wang, M., Liang, M., Yang, X., Xu, X., Zou, H., and Qiu, J. (2011). Activation of Sirt1 by resveratrol inhibits TNF-α induced inflammation in fibroblasts. PLoS One *6*, e27081. https://doi.org/10.1371/journal.pone.0027081.

Zhou, Y., Zhou, B., Pache, L., Chang, M., Khodabakhshi, A.H., Tanaseichuk, O., Benner, C., and Chanda, S.K. (2019). Metascape provides a biologist-oriented resource for the analysis of systems-level datasets. Nat. Commun. *10*, 1523. https://doi.org/10.1038/s41467-019-09234-6.

STAR★METHODS

KEY RESOURCES TABLE

REAGENT or RESOURCE	SOURCE	IDENTIFIER
**Antibodies**		
Rabbit mAb anti-CDX2 (D11D10)	Cell Signaling Technology	Cat# 12306; RRID: AB_2797879
Goat Polyclonal anti-SOX2	Bio-Techne	Cat# AF2018-SP; RRID: AB_355110
Rabbit Polyclonal anti-Cleaved Caspase-3	Cell Signaling Technology	Cat# 9661; RRID: AB_2341188
Rabbit Polyclonal anti-ZO1	Proteintech	Cat# 21773-1-AP; RRID: AB_2878956
Rabbit mAb anti-Oct4	Abcam	Cat# ab181557; RRID: AB_2892708
Mouse Monoclonal anti-TFAP2C [3B5]	Abcam	Cat# ab110635; RRID: AB_10861449
Mouse Monoclonal anti-E Cadherin [M168]	Abcam	Cat# ab76055; RRID: AB_1310159
Donkey anti-Rabbit IgG H&L (Alexa Fluor 488)	Abcam	Cat# ab150073; RRID: AB_2632917
Donkey anti-Rabbit IgG H&L (Alexa Fluor 647)	Abcam	Cat# ab150075; RRID: AB_2752244
Donkey anti-Rabbit IgG H&L (Alexa Fluor 555)	Abcam	Cat# ab150074; RRID: AB_2810263
Donkey anti-Mouse IgG H&L (Alexa Fluor 488)	Abcam	Cat# ab150105; RRID: AB_2732856
Donkey anti-Mouse IgG H&L (Alexa Fluor 647)	Abcam	Cat# ab150107; RRID: AB_2687532
Donkey anti-Mouse IgG H&L (Alexa Fluor 555)	Abcam	Cat# ab150106; RRID: AB_2810264
**Chemicals, peptides, and recombinant proteins**		
Advanced DMEM/F12	Gibco	Cat# 12634010
Collagenase Type IV	Gibco	Cat# 17104019
Fetal Bovine Serum (FBS)	Gibco	Cat# A5669701
DMEM/F12	Gibco	Cat# 21331020
L-glutamine	Gibco	Cat# 25030081
Trypsin-EDTA (0.05%)	Gibco	Cat# 25300062
Phenol red-free DMEM/F12	Gibco	Cat# 21041025
TrypLE Express	Gibco	Cat# 12604021
DPBS	Gibco	Cat# 14190144
N2 Supplement	Gibco	Cat# A1370701
B27 Supplement	Gibco	Cat# 12587010
Penicillin-Streptomycin (P/S)	Gibco	Cat# 15140122
GlutaMAX Supplement	Gibco	Cat# 25030081
HEPES	Gibco	Cat# 15630106
DNase I	Beyotime	Cat# D7076
Cytochalasin D	Beyotime	Cat# Y268078

(Continued on next page)

Continued

REAGENT or RESOURCE	SOURCE	IDENTIFIER
Recombinant Human FGF10	PeproTech	Cat# 100-26
Recombinant Human HGF	PeproTech	Cat# 100-39
Recombinant Human/Mouse Activin A	PeproTech	Cat# 120-14
Thiazovivin	Selleck	Cat# S1459
GSK429286	Selleck	Cat# E1556
N-acetyl-L-cysteine	MCE	Cat# HY-134495
8-bromo-cAMP	MCE	Cat# HY-B1511
β-Estradiol	MCE	Cat# HY-B0141
Y-27632	MCE	Cat# HY-10071
Recombinant Mouse Prolactin	MCE	Cat# HY-P70029
XAV939	MCE	Cat# HY-15147
Wortmannin	MCE	Cat# 19545-26-7
LY294002	MCE	Cat# HY-10108
PP2	MCE	Cat# HY-13805
Z-VAD-FMK	MCE	Cat# HY-16658B
Recombinant Mouse IGF-1	MCE	Cat# HY-P7018
Resveratrol	MCE	Cat# HY-16561
Blebbistatin	MCE	Cat# HY-13813
L-Ascorbic acid 2-phosphate magnesium	MCE	Cat# HY-103701A
Penicillin-Streptomycin (100×), Sterile	MCE	Cat# HY-K1006
Sodium bicarbonate	MCE	Cat# HY-Y0756
Pyruvic acid sodium	MCE	Cat# HY-W015913
Recombinant Mouse LIF	Millipore	Cat# LIF1050
SPY650-FastAct	Cytoskeleton	Cat# CY-SC505
TRNzol Universal reagent	Tiangen	Cat# DP424
All-In-One 5× RT MasterMix	Applied Biological Materials	Cat# G490
TB Green Premix Ex Taq II (Tli RNaseH Plus)	Takara	Cat# RR820A
Tissue-Tek O.C.T. Compound	Sakura	Cat# 4583
RPMI 1640 base medium	Thermo Fisher Scientific	Cat# 11875093
Tween 20 Surfact-Amps	Thermo Fisher Scientific	Cat# 85113
Triton X-100 Surfact-Amps	Thermo Fisher Scientific	Cat# 85111
UltraPure Glycine	Thermo Fisher Scientific	Cat# 15527013
Lyso-Tracker Red DND-99	Thermo Fisher Scientific	Cat# L7528
DAPI Nucleic Acid Stain	Thermo Fisher Scientific	Cat# D3571
Human FGF-4	R&D Systems	Cat# 235-F4

(*Continued on next page*)

Continued		
**REAGENT or RESOURCE**	**SOURCE**	**IDENTIFIER**
**Deposited data**		
Raw and analyzed RNA-seq data	This paper	PRJCA067470
**Experimental models: cell lines**		
Mouse: GFP-labeled ESC lines	This paper	Derived from CAG-GFP X 129S1
Mouse: GFP-labeled TSC lines	This paper	Derived from CAG-GFP X 129S1
Mouse: GFP-labeled PrESC lines	This paper	Derived from CAG-GFP X 129S1
Mouse: mCherry-labeled ITS lines	This paper	Derived from wild-type ICR strain
Mouse: mCherry-labeled E65TS lines	This paper	Derived from wild-type ICR strain
**Software and algorithms**		
R (version 4.1.2)	R Core Team	https://www.r-project.org/
Prism 8	GraphPad	https://www.graphpad.com/features
ImageJ/Fiji	Schindelin et al., 2012	https://fiji.sc/
Trim Galore (version 0.6.7)	Krueger et al., 2021	https://www.bioinformatics.babraham.ac.uk/projects/trim_galore/
STAR aligner (version 2.7.9a)	Dobin et al., 2013	https://github.com/alexdobin/STAR
featureCounts	Liao et al., 2014	http://subread.sourceforge.net/
DESeq2	Love et al., 2014	https://bioconductor.org/packages/release/bioc/html/DESeq2.html
Metascape	Zhou et al., 2019	https://metascape.org/
**Other**		
40 μm cell strainer	BIOLOGIX	Cat# 15-1040
70 μm cell strainer	BIOLOGIX	Cat# 15-1070
FACS tube	BD	Cat# 352054
24 mm Transwell (8.0 μm pore)	Corning	Cat# 3428

EXPERIMENTAL MODEL AND STUDY PARTICIPANT DETAILS

Animals

All animal experiments were approved by the Institutional Animal Care and Use Committee of Tongji University and were performed in accordance with institutional guidelines.

Stem cell lines

GFP-labeled ESC, TSC, and PrESC lines used in this study were derived from E3.5 blastocysts generated by crossing homozygous CAG-GFP transgenic males (C57BL/6 background) with 129S1 females. To validate the generality of TSC elimination, additional mCherry-labeled ITS and E65TS lines derived from wild-type ICR embryos were included. E65TS cells were maintained in feeder-free TXV medium. TSC/ITS Lines (female): Derived from E3.5 blastocysts, the embryos were cultured on MEF feeders in TS medium until the trophectoderm (TE) outgrowths appeared. TSCs were maintained in TS medium consisting of RPMI 1640 supplemented with 20% FBS, 25 ng/mL FGF4, 1 μg/mL heparin, and 70% MEF-conditioned medium. E65TS Lines (male): Derived from the extra-embryonic ectoderm (ExE) of E6.5 embryos. The ExE was microsurgically isolated from the epiblast and ectoplacental cone using fine tungsten needles.

Isolated ExE fragments were initially cultured on MEF feeders in complete chemically defined, serum-free TXV medium (DMEM/F-12 supplemented with 200 μM ASAP, 1 mM sodium pyruvate, 1% GlutaMAX, 10 mM HEPES, and 100 pM β-mercaptoethanol, 25 ng/mL Human FGF4, 2 ng/mL Human TGF-β, 1 μg/mL Heparin, 50 ng/mL Activin A, 50 ng/mL Murine IL-11, 25 ng/mL Human BMP7, 200 μM 8-bromo-cAMP, and 5 nM 1-Oleoyl lysophosphatidic acid (LPA). For downstream feeder-free adaptation, stable cell colonies were subsequently transitioned at passage P3 to P4 onto culture plates pre-coated with 5 μg/mL Human Recombinant Laminin 521 and maintained in TX medium formulation consisting of DMEM/F12 without HEPES and L-glutamine, 64 mg/L L-ascorbic acid-2-phosphate magnesium, 14 μg/L sodium selenite, 19.4 mg/L insulin, 543 mg/l $NaHCO_3$, 10.7 mg/L holo-transferrin, 25 ng/mL human recombinant FGF4, 2 ng/mL human recombinant TGFB1, 1 μg/mL heparin, 2 mM L-glutamine, and 1% penicillin-streptomycin.

Primitive Endoderm Stem Cells (PrESCs): PrESCs were established from E3.5 blastocysts by culturing them in CLBA medium on MEF feeders. The CLBA medium contained DMEM/F12 supplemented with 3 μM CHIR99021, 1000 U/mL LIF, 10 ng/mL bFGF, and 10 ng/mL Activin A. Cells were passaged every 3–4 days using 0.05% Trypsin-EDTA.

All cell lines were routinely tested for mycoplasma and confirmed to be negative.

METHOD DETAILS

Embryo collection and processing

Female B6D2F1 mice (6–8 weeks old) were superovulated by intraperitoneal injection of 5 IU pregnant mare serum gonadotropin (PMSG), followed 48 h later by 7 IU human chorionic gonadotropin (hCG), and subsequently mated with B6D2F1 males. Eight-cell embryos were collected and cultured in G1-PLUS medium at 37°C in 5% CO2 until further manipulation.

Chimera embryo production

To generate chimeric embryos, donor stem cells (TSCs, ESCs, PrESCs, ITS or E65TS between passages 10 and 20) were dissociated, washed with PBS, and pre-plated on 35 mm dishes for 10 minutes to deplete MEF feeders. The non-adherent cells were collected, centrifuged, and maintained on ice. Approximately 10-15 GFP-labeled donor cells were microinjected into each host 8-cell stage embryo. Following microinjection, the chimeric embryos were cultured in G1-PLUS medium supplemented with or without 2 μM Y-27632 (or other specified inhibitors) until the blastocyst stage, prior to *in vitro* analysis or *in vivo* embryo transfer.

Cell dissociation

Stem Cells were dissociated using 0.05% Trypsin-EDTA for 4 min. Cell suspensions were centrifuged (200 × g, 4 min), resuspended, and incubated on 0.1% gelatin-coated plates for 15–30 min to allow feeder attachment. Non-adherent cells were collected and stored on ice.

Transwell migration assay

To assess cell motility, donor TSCs (treated with or without ROCKi) were seeded in the upper chamber of 8 μm pore Transwell inserts. After 24 h of incubation, cells that migrated to the lower surface were fixed with 4% PFA and stained with 0.1% crystal violet for quantification. Migration efficiency was determined by counting five random fields per membrane.

RNA extraction and RT-qPCR

Total RNA was extracted using TRIzol reagent according to the manufacturer's instructions. cDNA synthesis was performed using 5× All-In-One RT MasterMix, and quantitative PCR was carried out using TB Green Premix Ex Taq II on an ABI7500 Real-Time PCR System. Data were analyzed using GraphPad Prism 8.

Immunofluorescence staining

Placentas collected at E12.5 were fixed overnight with 4% paraformaldehyde (Servicebio, China) at 4°C. After fixation, the placentas were embedded in paraffin and cut along the largest longitudinal cross-sections. The sections were stained with hematoxylin and eosin (H&E) and photographed with a microscope. The areas of the decidua, spongiotrophoblast layer and labyrinth layer were counted using ImageJ software. Blastocysts were fixed with 4% paraformaldehyde (Servicebio) overnight at 4°C. After fixation, the samples were permeabilized with 0.5% Triton X-100 (Sigma, Germany) for 5 min

at room temperature. Then, the samples were blocked with 3% bovine serum albumin (BSA)-PBS (MP Biomedicals, USA) for 1-2 h at room temperature. Samples were incubated with primary antibodies (1:500 in 3% BSA-PBS) overnight at 4°C, washed three times with PBS, then incubated with species-matched secondary antibodies (1:500 in 3% BSA-PBS, 1-2 h, RT). Nuclei were counterstained with DAPI (10 min, dark), mounted with antifade reagent, and imaged by confocal microscopy.

Smart-seq2 library preparation and sequencing

RNA libraries were prepared following the Smart-seq2 protocol (Picelli et al., 2014). cDNA was amplified for 15 cycles, purified with AMPure XP beads, and sequenced on the Illumina HiSeq platform. A Kapa Hyper Prep Kit (KAPA, KK8504, Switzerland) was used to complete the sequence libraries. The libraries were sequenced on Illumina HiSeq 2500, Illumina HiSeq X10 and Illumina NovaSeq instruments with paired-end reads of 125 bp or 150 bp. Quality control and sequencing were performed by Berry, Novogene and ANOROAD Genomics Corporation.

Live-cell imaging and internalization-event tracking

Chimeric embryos were placed in a 5% CO_2 incubator-integrated confocal microscope (Zeiss LSM 880). Images were captured every 40 s using a 20× or 40× water-immersion objective. The duration of each event was calculated from the initial contact (membrane invagination) to the complete disappearance of the donor cell into the host cytoplasm.

LysoTracker labeling

To visualize lysosomal degradation, chimeric embryos were incubated in G1-PLUS medium containing 50 nM Lyso-Tracker Red DND-99 (Thermo Fisher, L7528) for 30 minutes at 37°C prior to imaging. Colocalization of GFP-labeled donor TSC remnants and Lyso-Tracker Red signal within host blastomeres was assessed as evidence of localization to acidic intracellular compartments.

Karyotype analysis

Donor cell lines (TSC, ITS, E65TS) at passage 15–20 were treated with 0.1 μg/mL Colcemid (Gibco) for 4 hours at 37°C. Cells were then dissociated, incubated in 0.075 M KCl hypotonic solution for 20 minutes, and fixed with a methanol/acetic acid (3:1) solution. Metaphase spreads were prepared by dropping the cell suspension onto chilled glass slides and stained with Giemsa. At least 50 metaphase cells were counted for each cell line to determine the modal chromosome number. The modal chromosome number and the percentage of euploid metaphases are shown in Figure S2.

Pharmacological treatments and apoptosis induction

For apoptosis induction, donor TSCs were treated with 150 μM H2O2 for 12 h before embryo injection. For inhibitor screening, chimeric embryos were cultured in G1-PLUS medium containing Wortmannin (1 μM), LY294002 (10 μM), PP2 (10 μM), Z-VAD-FMK (20 μM), IGF-1 (100 ng/mL), Resveratrol (10 μM), Y-27632 (2–10 μM), GSK429286 (10 μM), Cytochalasin D (0.5–2 μM), or the indicated concentration of other compounds. Equivalent volumes of DMSO were used as vehicle controls. For donor-cell pretreatment experiments, TSCs were incubated with 2 μM Y-27632 or 500 nM Thiazovivin for 12 h before microinjection. For embryo-culture treatments, the indicated inhibitors were added immediately after donor-cell injection and maintained in the culture medium until the designated developmental stage.

Processing of E12.5 placentas for FACS

E12.5 placentas were dissected and the maternal decidua was carefully removed. The placental tissues were minced and digested with 0.25% Trypsin-EDTA and 1 mg/mL Collagenase Type IV (Gibco) for 30 minutes at 37°C. The resulting cell suspension was filtered through a 40 μm cell strainer, washed with 2% FBS-PBS, and analyzed for GFP expression using a MoFlo Astrios EQ flow cytometer.

Smart-seq2 analysis

RNA-seq reads were processed with FastQC and Trimmomatic, aligned to mm10 (STAR), and quantified (featureCounts). Differential expression analysis (DESeq2), GO enrichment (Metascape), and GSEA (MsigDB) were performed. Raw and analyzed RNA-seq data have been deposited in the Genome Sequence Archive (GSA) under accession number GSA: PRJCA067470.

Image analysis and quantification using ImageJ

All digital image processing and quantitative analyses were performed using ImageJ/Fiji software. To evaluate donor cell retention, the fluorescence area of GFP/mcherry TSCs was measured and expressed as a ratio of the total area of the chimeric embryo. For membrane dynamics analysis, the number of actin-rich protrusions at the host-donor interface was manually quantified from high-resolution confocal Z-stack images. Cell migration was assessed by counting crystal violet-stained cells on the lower surface of the Transwell membrane across five randomly selected microscopic fields per insert. For all fluorescence intensity measurements, background subtraction was performed to ensure quantitative consistency across experimental groups.

QUANTIFICATION AND STATISTICAL ANALYSIS

All statistical analyses were performed using GraphPad Prism 8. Data are presented as mean ± SD unless otherwise indicated. Statistical significance was determined using unpaired two-tailed Student's t test, one-way ANOVA with Tukey's multiple comparisons test, or two-way ANOVA as indicated in the figure legends. $p < 0.05$ was considered statistically significant. The numbers of embryos, biological samples, and independent experiments are indicated in the corresponding figure legends.

Stem Cell Reports, Volume *21*

Supplemental Information

ROCK inhibition suppresses host blastomere-mediated elimination and enhances trophoblast stem cell contribution in mouse chimeras

Baoxing Dong, Kuisheng Liu, Chenxiang Xi, Sheng Wang, Yifan Sheng, Jiani Xiang, Jiayu Chen, Yingdong Liu, Dandan Bai, Shanyao Liu, Xia Tu, Xiaochen Kou, Yanhong Zhao, Jiqing Yin, Hong Wang, Binya Liu, Changle Ma, Shaorong Gao, and Wenqiang Liu

Supplemental Information

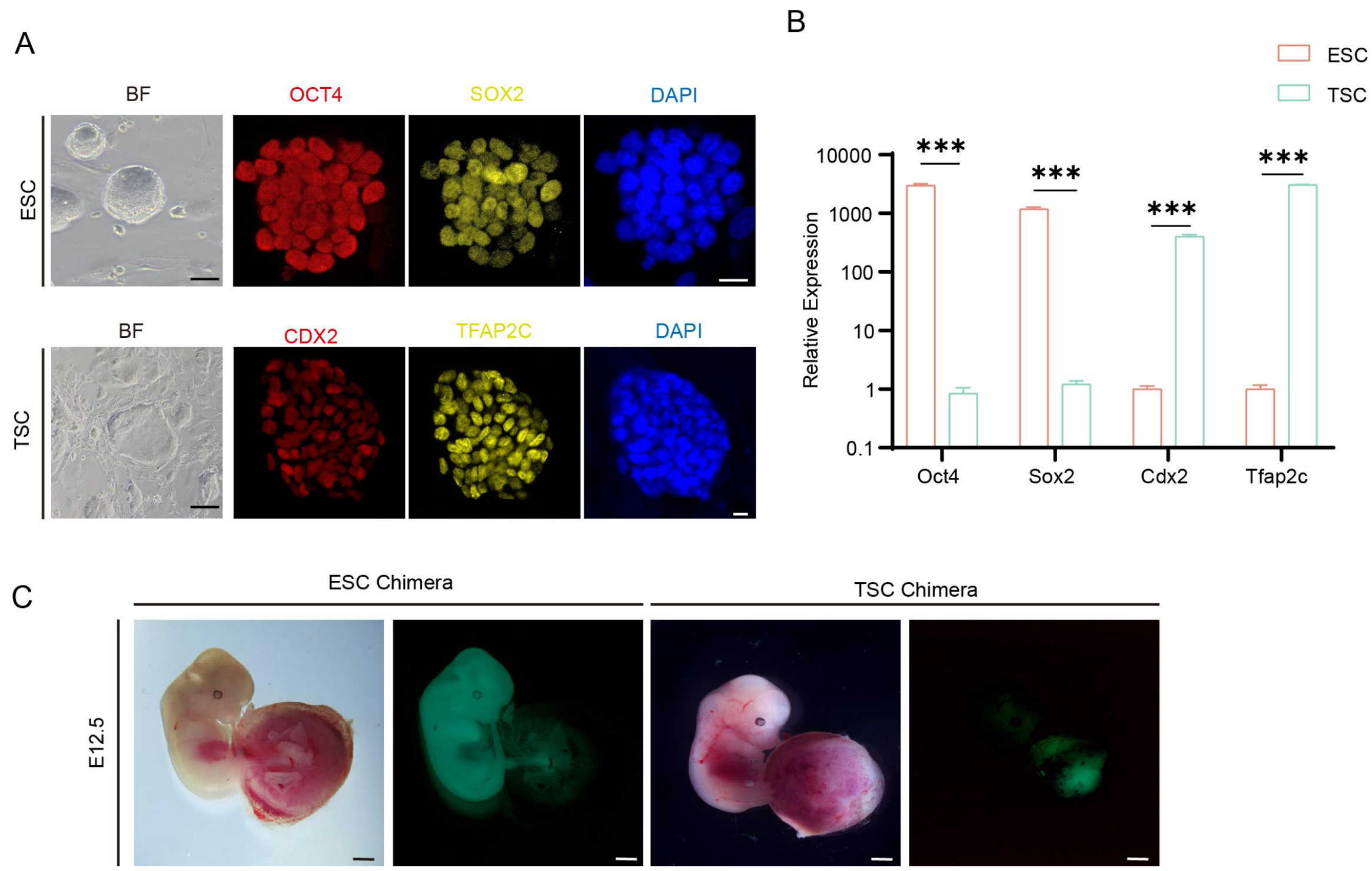

Figure S1. Characterization and In Vivo Contribution Potential of Derived Stem Cell Lines (Related to Figure 1)

(A) Immunofluorescence characterization of donor cell lines. ESC clones were stained for pluripotency markers OCT4 and SOX2; TSC clones were stained for trophoblast markers CDX2 and TFAP2C. Scale bar = 25 μm.

(B) Quantitative PCR (qPCR) analysis of pluripotency and trophoblast lineage-specific markers in derived ESC and TSC lines, with expression levels normalized to the internal reference gene Gapdh. Data are presented as mean ± SD. ***$P < 0.001$, determined by unpaired two-tailed Student's t-test. Data were analyzed using the $2^{\wedge -\Delta\Delta CT}$ method.

(C) Morphology of E12.5 chimeras. Representative bright-field and fluorescence images of embryos following ESC or TSC injection. Scale bar = 2 mm.

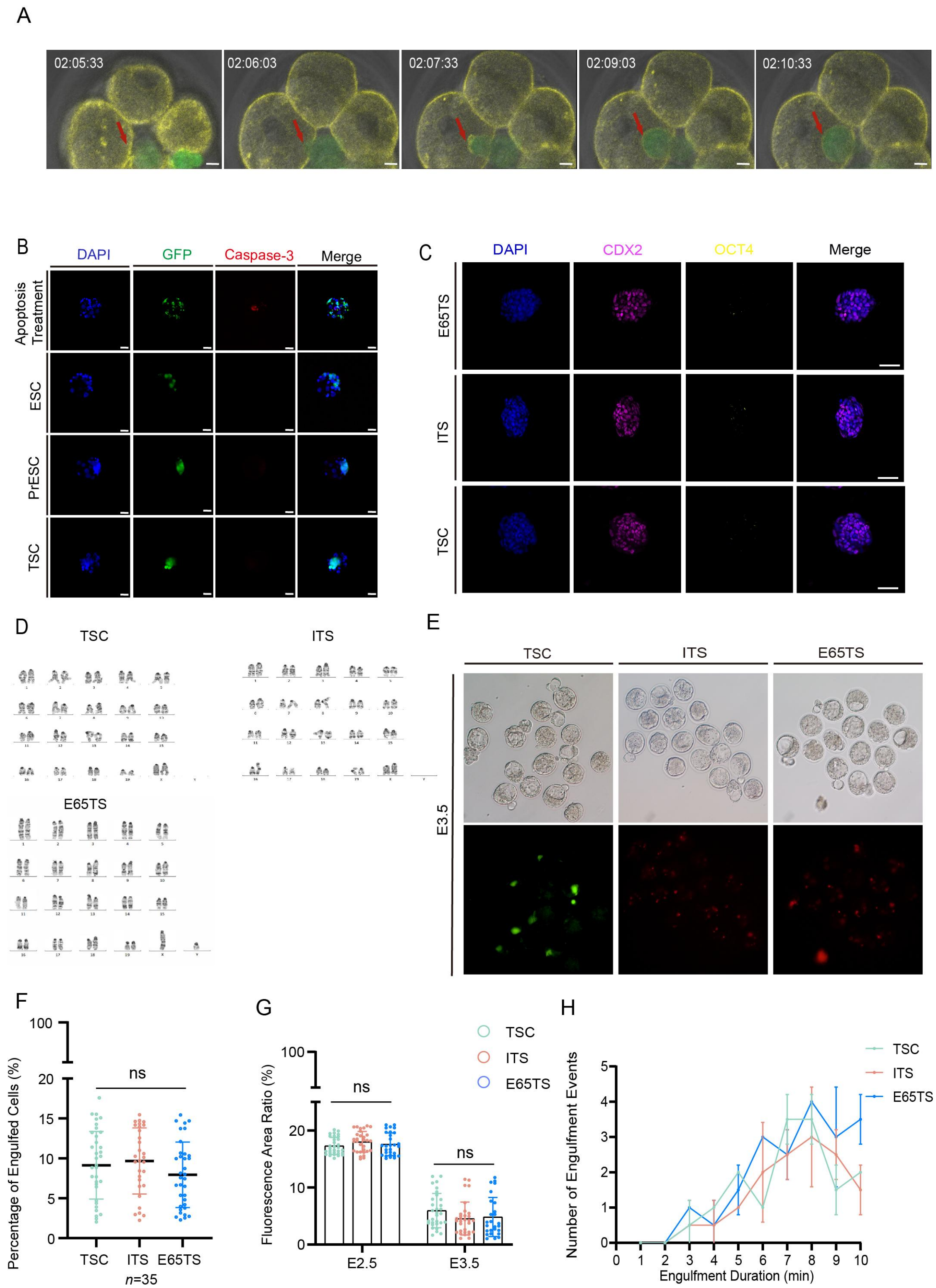
A
02:05:33
02:06:03
02:07:33
02:09:03
02:10:33
B
DAPI
GFP
Caspase-3
Merge
Apoptosis Treatment
ESC
PrESC
TSC
C
DAPI
CDX2
OCT4
Merge
E65TS
ITS
TSC
D
TSC
ITS
E65TS
E
TSC
ITS
E65TS
E3.5
F
Percentage of Engulfed Cells (%)
100
20
15
10
5
0
ns
TSC
ITS
E65TS
n=35
G
Fluorescence Area Ratio (%)
100
20
10
0
ns
ns
E2.5
E3.5
TSC
ITS
E65TS
H
Number of Engulfment Events
5
4
3
2
1
0
0 1 2 3 4 5 6 7 8 9 10
Engulfment Duration (min)
TSC
ITS
E65TS

Fig. S2 Characterization of TSC Internalization Across Multiple Cell Lines. (Related to Figure 2)

(A) Dynamics of phagocytic engulfment. Time-lapse imaging showing the active internalization of donor cells (green) by host blastomeres (yellow, actin). Red arrows indicate donor cells undergoing cell engulfment.

(B) Apoptosis assessment of chimeric embryos. Immunofluorescence staining for Caspase-3 in ESC, PrESC, and TSC chimeras at 4 hours post-injection, showing negligible apoptosis in donor cells. Scale bar = 5 μm.

(C) Immunofluorescence of diverse TSC lines. Characterization of ITS (pre-implantation origin) and E65TS (post-implantation origin) lines using CDX2 and OCT4 markers to confirm TSC identity.

(D) Karyotype analysis. Representative karyotypes of TSC, ITS, and E65TS lines, demonstrating chromosomal stability.

(E) Representative brightfield and fluorescence images of chimeric embryos at the late blastocyst (E3.5) stage.

(F) Representative images of chimeric blastocysts. Comparison of engulfment events among TSC, ITS, and E65TS donor groups at E3.5. Data are presented as mean ± SD. not significant, determined by one-way ANOVA followed by Tukey's post-hoc test. *n*=42 for TSC, 39 for ITS, and 45 for E65TS (*N*=3 independent experiments).

(G) Quantitative analysis of TSC line engulfment. Statistics showing the percentage of engulfed cells and the fluorescence area ratio for TSC, ITS, and E65TS at the morula (E2.5) and blastocyst (E3.5) stages. Data are presented as mean ± SD. ns, not significant, determined by one-way ANOVA followed by Tukey's post-hoc test. *n*=35 for TSC, 37 for ITS, and 32 for E65TS (*N*=3 independent experiments).

(H) Time distribution statistics of phagocytic events. The number distribution of phagocytic events occurring in the three cell lines (TSC, ITS, and E65TS) at different time points. Temporal distribution and duration of engulfment events across different

TSC lines. Data were recorded from N=20 embryos and processed using GraphPad Prism.

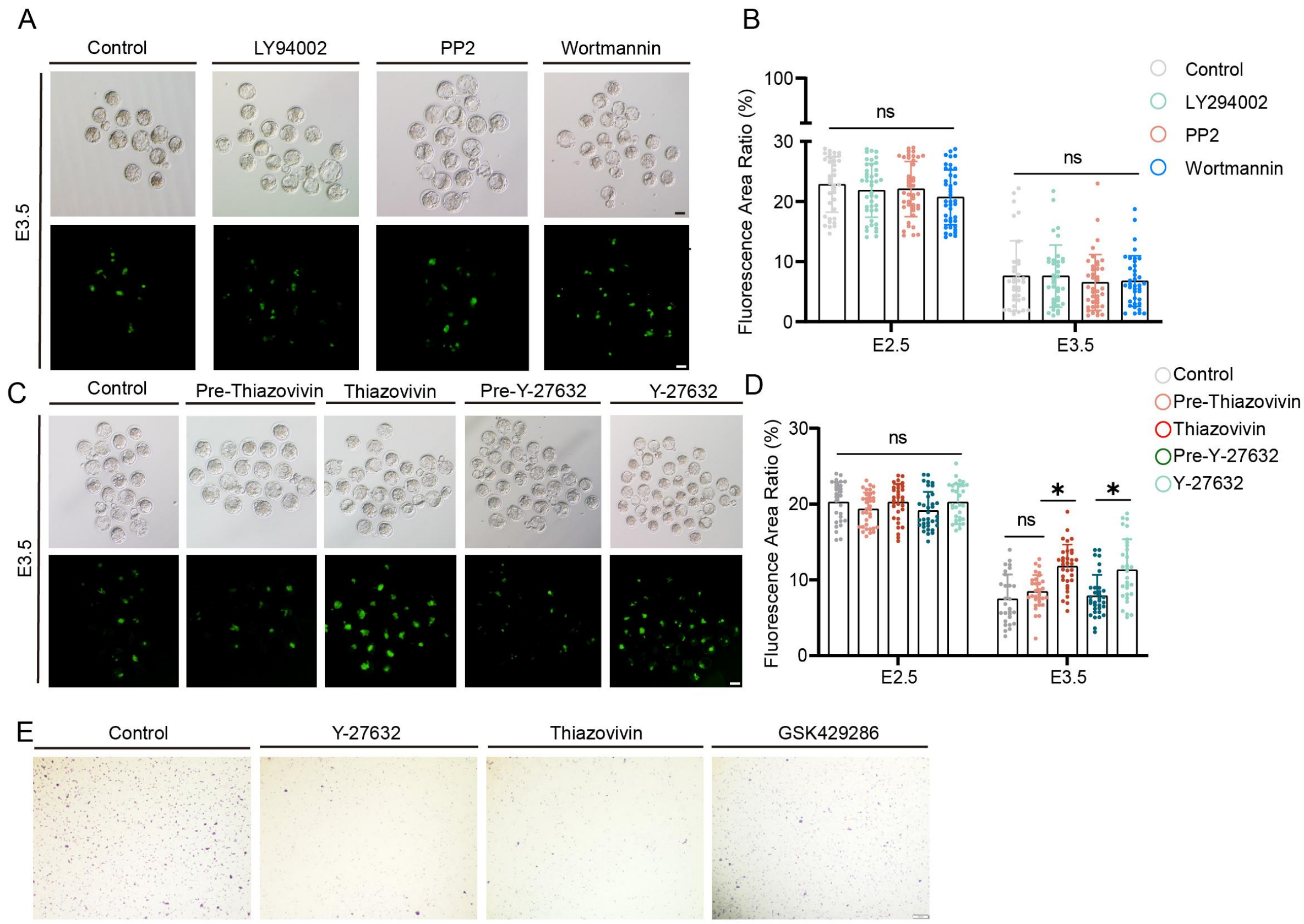

Figure S3. Pharmacological Screening and Treatment Strategies Comparison (Related to Figure 3)

(A) Pharmacological screening for phagocytosis inhibition. Representative fluorescence images of E3.5 embryos treated with PI3K inhibitors (Wortmannin, LY294002) and Src family kinase inhibitor (PP2). Scale bar = 100 μm

(B) Statistics of cell retention. Fluorescence area ratio (%) at E2.5 and E3.5 stages under different phagocytosis inhibitors, showing limited rescue effects compared to control. Statistical analysis of cell retention under various phagocytosis inhibitors. Data are presented as mean ± SD. ns, not significant, determined by one-way ANOVA followed by Tukey's post-hoc test. *n*=36 for Control, 41 for Wortmannin, 42 for LY294002, and 40 for PP2 (*N*=3 independent experiments).

(C) Comparison of pre-treatment vs. co-culture treatment. Representative images showing the effect of pre-incubating donor TSCs with ROCK inhibitors (Pre-Thiazovivin,

Pre-Y-27632) compared to active treatment during embryo chimerism.

(D) Quantitative statistics of treatment timing. Statistics of fluorescence area ratio at E2.5 and E3.5. Data are presented as mean ± SD. $^{**}P < 0.01$, determined by one-way ANOVA followed by Tukey's post-hoc test. n=32 for Control, 34 for Pre-Thiazovivin, 32 for Thiazovivin, 35 for Pre-Y-27632, and 31 for Y-27632 (N=3 independent experiments).

(E) Transwell migration assay. Quantification and representative images showing that ROCK inhibition impairs the migratory capability of TSCs. Scale bar =50 m

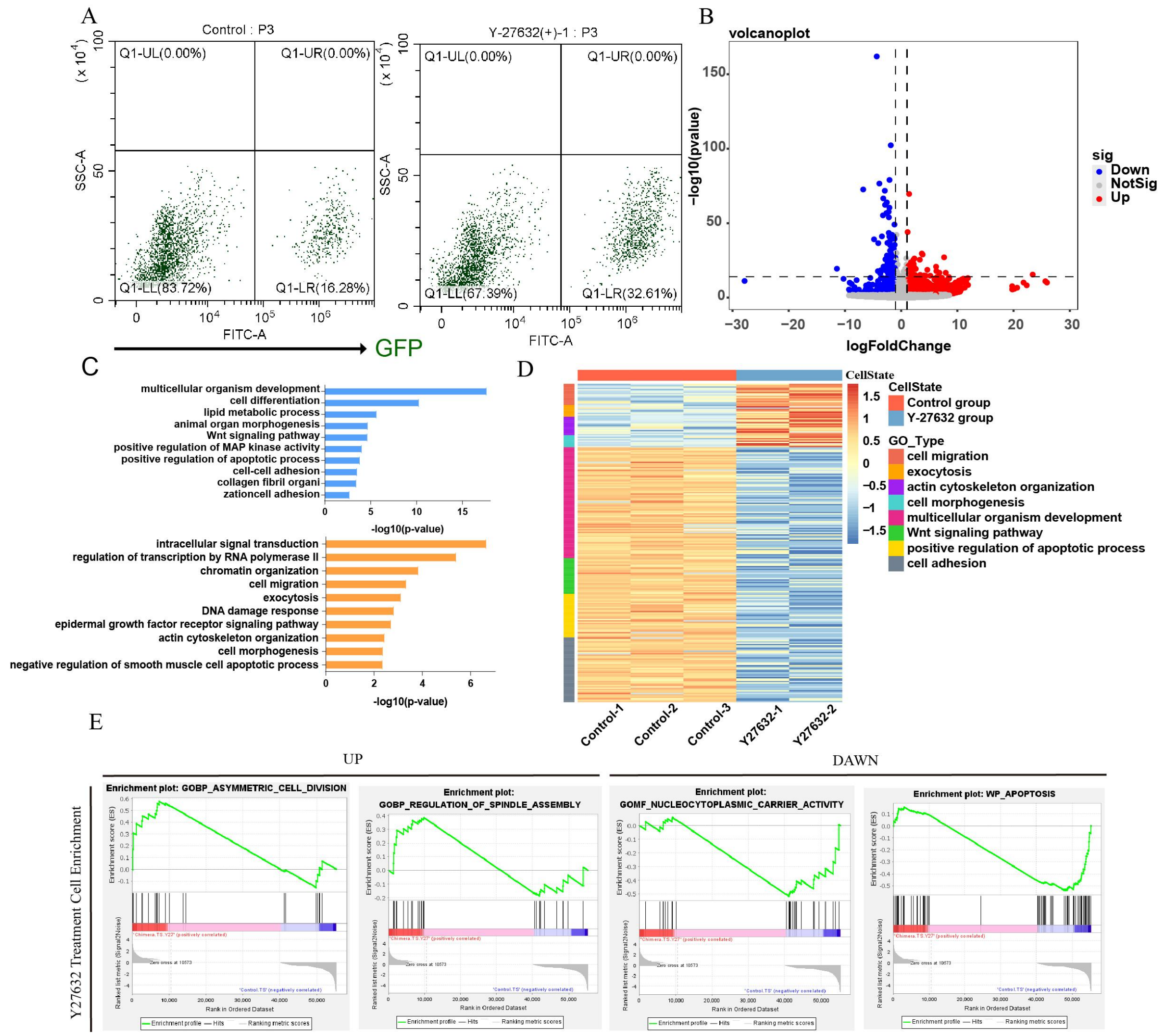

Figure S4. Transcriptomic Profiling and Long-term Flow Cytometry Validation. (Related to Figure 4)

(A) Flow cytometry (FACS) analysis of E12.5 placentas. Representative FACS plots showing the proportion of GFP-positive donor cells in the Y-27632-treated group versus the control group.

(B) Volcano plot of differentially expressed genes (DEGs). Comparison of gene expression between Y-27632-treated and control morula-stage TSCs ($|\log2FC| > 1$, FDR < 0.05).

(C) Gene Ontology (GO) enrichment analysis. Functional annotation of DEGs highlighting pathways involved in actin cytoskeleton organization, cell migration, and

positive regulation of the apoptotic process.

(D) Cluster heatmap of functional genes. Expression profiles of genes associated with cytoskeletal remodeling, cell adhesion, and exocytosis in control versus Y-27632 treated groups.

(E) Gene Set Enrichment Analysis (GSEA). Enrichment plots for asymmetric cell division, regulation of spindle assembly, and apoptosis pathways following ROCK inhibition
